# Day-by-Day Ambient Determination of Nighttime Heterogeneous Oxidation of Particle-Phase Benzo[a]pyrene in an Urban Environment

**DOI:** 10.1021/acs.est.6c02669

**Published:** 2026-04-07

**Authors:** Qiongqiong Wang, Zongjun Li, Zhongliang Huang, Shuhui Zhu, Jie Li, Liping Qiao, Min Zhou, Dandan Huang, Hongli Wang, Qingyan Fu, Huan Yu, Jian Zhen Yu

**Affiliations:** † Department of Atmospheric Science, School of Environmental Studies, 12564 China University of Geosciences, Wuhan 430074, China; ‡ Laboratory of Formation and Prevention of Urban Air Pollution Complex, Ministry of Ecology and Environment, 177494 Shanghai Academy of Environmental Sciences, Shanghai 200233, China; § Institute of Molecular Aggregation Science, 12605 Tianjin University, Tianjin 300072, China; ∥ Department of Chemistry, Hong Kong University of Science and Technology, Hong Kong 999077, China

**Keywords:** PAHs, benzo[a]pyrene, heterogeneous oxidation, decay rates, online measurements, urban environments

## Abstract

Benzo­[a]­pyrene (BaP) is widely used as a model compound for studying the heterogeneous oxidation of particle-borne polycyclic aromatic hydrocarbons (PAHs), yet its decay kinetics under real atmospheric environments remain poorly constrained. Here, we present the first day-by-day determination of nighttime decay rates of particulate BaP using bihourly measurements in urban Shanghai. Two decay patterns were observed: incomplete decay with substantial remaining BaP, and complete decay with little residual BaP. Across 88 nights, decay rates averaged 0.21 ± 0.15 h^–1^, with 0–87% of BaP remaining unreacted by the end of night. Decay rates increased linearly with gas-phase ozone and showed a temperature-dependent relationship with initial BaP concentration across three temperature regimes (TR_1_: 5–16 °C; TR_2_: 16–25 °C; TR_3_: 25–30 °C). Effective activation energies differed markedly between those of TR_1–2_ and TR_3_. Residue BaP was negatively correlated with relative humidity across all regimes, whereas its positive correlation with organic carbon mass was confined to TR_1_ and absent in TR_3_, suggesting a transition in the aerosol phase state. Nighttime atmospheric lifetimes of BaP were 5.2, 9.3, and 13.1 h under TR_1_, TR_2_, and TR_3_, respectively. These ambient constraints provide needed kinetic parameters for modeling the fate, transport, and health impacts of atmospheric PAHs.

## Graphical abstract

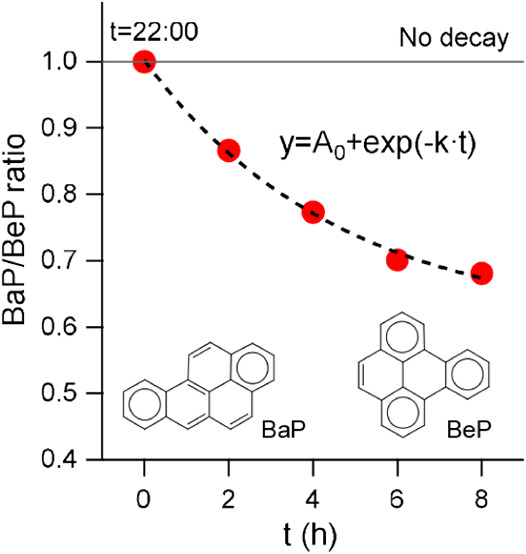

## 1 Introduction

Polycyclic aromatic hydrocarbons (PAHs), a large group of organic compounds with two or more fused benzene rings, are ubiquitous in the atmosphere. Low molecular weight PAHs with 2–3 benzene rings are mainly present in the gas phase, while heavier PAHs (with 4 or more aromatic rings) are usually associated with the particulate phase. Many PAHs are regarded as carcinogens, mutagens, and teratogens due to their ability to bind to DNA, posing a threat to human health upon chronic exposure.^1^ Besides, PAHs are key brown carbon (BrC) constituents and contribute significantly to BrC absorption, thus affecting aerosols’ radiative forcing.^2,3^ PAHs can react with atmospheric oxidants and transform into their derivatives, such as oxy-PAHs and nitro-PAHs, which could have even higher toxicities.^4^ Extensive efforts have been made to characterize the sources, properties, fates, and physical and chemical transformations of PAHs in the atmosphere.^5,6^ While gas-phase reactions of 2–3-ring PAHs with atmospheric oxidants have been extensively examined with generally consistent rate coefficients, heterogeneous reactions of heavy PAHs (>4-ring) remain less explored. These reactions exhibit higher complexity due to their dependence on substrate types and reaction conditions, resulting in substantially divergent estimates of decay rates across studies.^5^ Due to the lack of reliable kinetic data, the heterogeneous reactivity of PAHs has frequently been overlooked in modeling studies, hindering accurate assessment of the atmospheric fate of heavy PAHs.

Benzo­[a]­pyrene (BaP), bearing five fused benzene rings, is one of the most toxic species among the list of 16 priority PAHs by the U.S. Environmental Protection Agency (EPA)^7^ and is widely used as a representative PAH. Due to its low vapor pressure, BaP resides mostly in the aerosol phase. Heterogeneous oxidation of BaP by oxidants such as ·OH, ·NO_3_, and O_3_ is a major atmospheric loss pathway prior to deposition.^5^ The reaction kinetics are strongly affected by the aerosol matrix. For example, faster decay rates of BaP on soot particles than silica particles were reported.^8^ Burial effects of organic coatings on heterogeneous reactions of BaP with ozone were reported by experimental studies;^9^ phase separation and slow diffusion can significantly prolong the chemical lifetime of BaP,^10^ affecting long-range transport of PAHs in the atmosphere. A linear dependence^11,12^ and a nonlinear Langmuir-type dependence^13,14^ of the pseudo-first-order decay rate coefficient of BaP with gas-phase ozone concentrations were reported. However, previous studies predominantly investigated BaP adsorbed onto single-component surfaces, whereas atmospheric aerosol particles exhibit far greater complexity, typically consisting of both organic materials and inorganic salts with diverse morphologies under different atmospheric conditions. The evolution of matrix effects and the interplay between nonideality and phase state during multiphase chemical interactions, along with their collective impact on the environmental fate and long-range transport of BaP, remain inadequately characterized.

Diagnostic ratios of PAH pairs are well-established tools for distinguishing emission sources and assessing atmospheric aging.^15,16^ The ratio of BaP to its structural isomer benzo­[e]­pyrene (BeP) is particularly valuable in this context. Due to its higher reactivity, BaP degrades more rapidly than BeP during atmospheric transportation, causing the BaP/BeP ratio to decrease with aging.^17^ Consequently, this ratio serves as a useful indicator of atmospheric processing, with lower values consistently observed in aged aerosols from offline filter-based studies.^18–21^ The recent advent of online measurement techniques, specifically the Thermal-desorption Aerosol Gas chromatography and mass spectrometry (TAG), enables continuous hourly or bihourly measurements of individual PAH species.^22^ Recently, online bihourly measurement of other aerosol organics, such as oleic acid and levoglucosan, has proven effective in determining their ambient decay kinetics.^23,24^ Similarly, the high-time resolution measurement of PAHs by TAG could provide a potential tool to derive previously inaccessible quantitative decay kinetics for these compounds under real-world conditions.

In this study, a 7-month field campaign was conducted at an urban site in Shanghai, China, from March to September 2020. Bihourly measurements of PAHs were obtained by TAG. Using bihourly isomer-pair measurements, we quantified nighttime BaP decay rates under real atmospheric conditions for the first time. The influence of key environmental factors on the decay kinetics was investigated. The same analytical technique and kinetic approach can be extended to other PAH pairs to explore their ambient decay kinetics, providing essential kinetic parameters needed to improve the model predictions of PAH fate and transport.

## 2 Materials and Methods

### 2.1 Sampling and Chemical Analysis

Bihourly particle-phase concentrations of PAHs, including BaP and its isomers, were measured using the TAG system at an urban site in Shanghai, China, from March 1 to September 22, 2020. Details about the sampling site and PM pollution characterization can be found in our previous publications.^22,25^ The TAG system collected one-hour integrated samples at every even hour (e.g., 2:00 a.m., 4:00 a.m.), which underwent online thermal desorption and in situ derivatization prior to separation and detection by GC/MS. Quality assurance procedures for the measurement data used in this study are provided in Text S1. Concurrent measurements of major PM chemical compositions, including organic carbon (OC), were also available at this site. The meteorological parameters (temperature, T; relative humidity, RH; wind speed; and wind direction) and gaseous pollutants (O_3_, SO_2_, CO, NO, and NO_2_) were measured by collocated instruments at the site.

### 2.2 Relative Rate Constant Approach for BaP

Both experimental and modeling studies have established that the decay kinetics of condensed-phase PAHs frequently follow a rapid initial loss of PAHs in the first several hours and then a much slower decay at longer exposure times, leaving a nondepletable residual fraction.^10,26^ The heterogeneous reaction of BaP with oxidants is a bimolecular reaction. Its decay can be modeled using a pseudo-first-order kinetic framework, described by an exponential decay function incorporating a residual term (A_0_ ≥ 0) to account for the unreacted fraction.^10,27^

In this study, we adopted the relative rate constant approach to determine the ambient pseudo-first-order rate constant (*k*) for BaP. This method has been applied to calculate the ambient decay rates of unsaturated fatty acids such as oleic acid^23^ and levoglucosan.^24^ The key process was to select the reference compound (*j*) that shares common emission sources with the target species (*i*) but exhibits a significantly lower chemical reactivity. Thus, the temporal change in the concentration ratio $\left(\frac{C_{i}}{C_{j}}\right)$ directly reflects the decay kinetics of the target species *i*, under the assumption of no interference from fresh emissions. The specific expression as applied to ambient BaP is given by eq 1. We provide a residual term (A_0_) that represents the fraction of unreacted BaP during the studied time window. The detailed derivation of this equation is provided in Text S2. Given the complexity of the actual atmospheric environment, the above assumption is applicable only to days that can be adequately represented by the decay function. Variability in source emissions and the heterogeneity of the aerosol matrix may introduce additional uncertainties into the derived decay rates.

1 $$\frac{C_{i}}{C_{j}}=A_{0}+A_{1}\cdot e^{-k\cdot t},,k\approx k_{r_{i}}\cdot C_{\mathrm{ox}}$$

The physical and chemical properties of benzenoid hydrocarbons are discussed by Clar’s aromatic π-sextet rule^28^ and HOMO–LUMO energy gap,^29^ which posit that isomers with more sextets and larger HOMO–LUMO gaps are more stable than their counterparts. The Clar structures of BaP and its five commonly detected isomers (i.e., benz­[b]­fluoranthene, BbF; benz­[k]­fluoranthene, BkF; benz­[a]­fluoranthene, BaF; BeP; and perylene, PER) in atmospheric samples are shown in Figure 1a. The HOMO–LUMO energy gap of each isomer was further calculated using density functional theory and is shown in Figure 1b. BeP and BbF have three Clar π-sextets and a larger HOMO–LUMO gap (4.03 eV) than BkF, BaP, BaF, and PER, which have only two Clar π-sextets and lower HOMO–LUMO gaps (3.07–3.71 eV), implying higher intrinsic stability for BeP and BbF. We therefore selected BeP as the reference compound and additionally evaluated BbF as an alternative reference in the later analysis.

**Figure 1:**
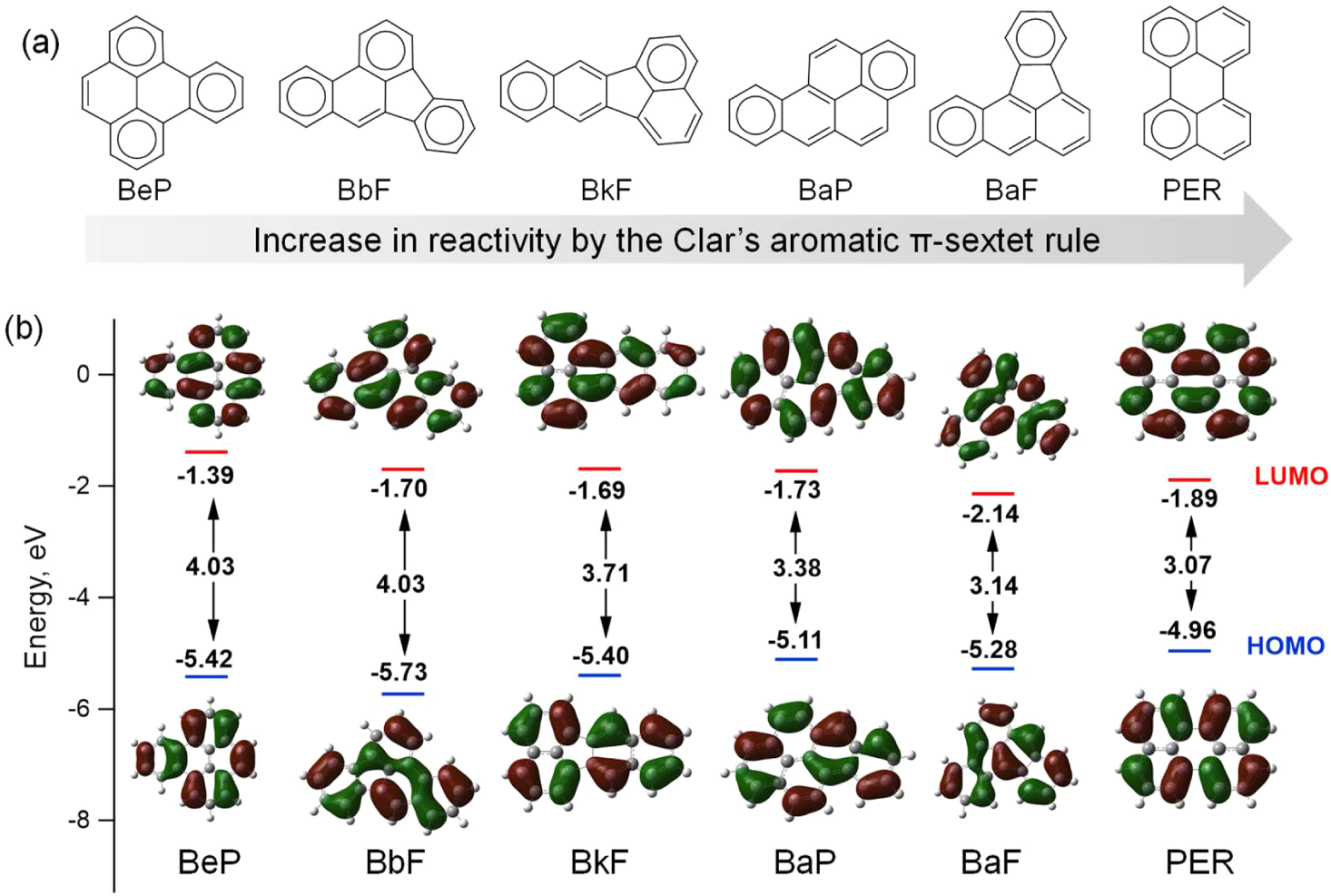
(a) Clar structures of BaP and its five isomers; aromatic π-sextets are indicated with circles. (b) HOMO and LUMO energies calculated by density functional theory for BaP and its isomers; the frontier molecular orbital distributions are shown, with red indicating the positive phase of the wave function and green indicating the negative phase.

Because BkF and PER showed low peak intensity and coelution problems in the ambient samples (Figure S4), leading to higher quantification uncertainties, we focus on decay kinetics of BaP and its reactive isomer, BaF. Laboratory studies have demonstrated that the heterogeneous oxidation of PAHs by ozone and nitrate radical is highly structure-dependent, with BaP exhibiting substantially higher reactivity than BeP,^17,30^ whereas the heterogeneous oxidation of PAHs by ·OH radicals shows weaker dependence on PAH structures.^31^ To minimize interference from ·OH chemistry, we restrict the analysis to nighttime data (22:00–06:00). We further assume negligible significant fresh emissions during this 8-h time window or that the variation of the concentration ratio was dominated by chemical reactions. Under this assumption, the decrease in the BaP/BeP ratio can be attributed to the chemical degradation of BaP. Given atmospheric complexity, these assumptions are expected to hold only on a subset of nights. Accordingly, we derive the ambient kinetic data for sampling days that satisfy these assumptions.

## 3 Results and Discussion

### 3.1 Overview of Ambient PAH Concentrations at the Observational Site

Figure 2a presents the temporal variations of measured BaP, BaF, and BeP concentrations, the BaP/BeP and BaF/BeP ratios, ozone levels, and key meteorological parameters over the campaign period. Average temperature and RH across the campaign period were 20.8 ± 6.6 °C and 76.6 ± 22.6%, ranging 6–30 °C and 40–100%, respectively. Average O_3_ and NO_2_ concentrations were 28.2 ± 13.4 and 18.2 ± 9.64 ppb, respectively. Temporal variations and statistical summaries of measured individual PAHs are shown in Figure S5 and Table S1. The total PAH concentration during the campaign ranged from 0.21 to 20.2 ng m^–3^, with an average value of 4.02 ± 2.38 ng m^–3^. This is much lower than levels reported for the heating season in 2020 in urban Beijing (67.7 ng m^–3^).^32^ Interspecies correlations of individual PAHs showed moderate to good correlations (Figure S6). The temporal profiles of BaP, BaF, and BeP were highly consistent (R_p_: 0.87–0.93; Figure S7), with elevated concentrations in March-April and August-September, and the lowest concentrations in June-July, suggesting common emission sources of the target PAH species. Average concentrations of BaP and BaF were 0.20 ± 0.17 ng m^–3^ and 0.05 ± 0.05 ng m^–3^, respectively, together accounting for 6.2% of the measured total PAHs. The BaP level is lower than previous observations at this site in winter in 2018 (0.48 ± 0.56 ng m^–3^).^22^ The concentration of BeP ranged from being not detected (ND) to 2.15 ng m^–3^, with an average value of 0.27 ± 0.21 ng m^–3^. The BaP/BeP and BaF/BeP ratios ranged from ND to 1.51 and from ND to 0.58, with average values of 0.69 ± 0.22 and 0.19 ± 0.08, respectively. Highest ratios were observed in March-April while the lowest ratios were observed in June-July, suggesting enhanced aging in summer months. The observations are in agreement with previous studies.^33^ Source apportionment analysis using the receptor model suggested that BaP, BaF, and BeP were predominantly from regional sources such as coal combustion and biomass burning (Figure S10), consistent with previous studies in this region.^25^ Detailed methodology and results of the source apportionment are provided in Text S3.

**Figure 2:**
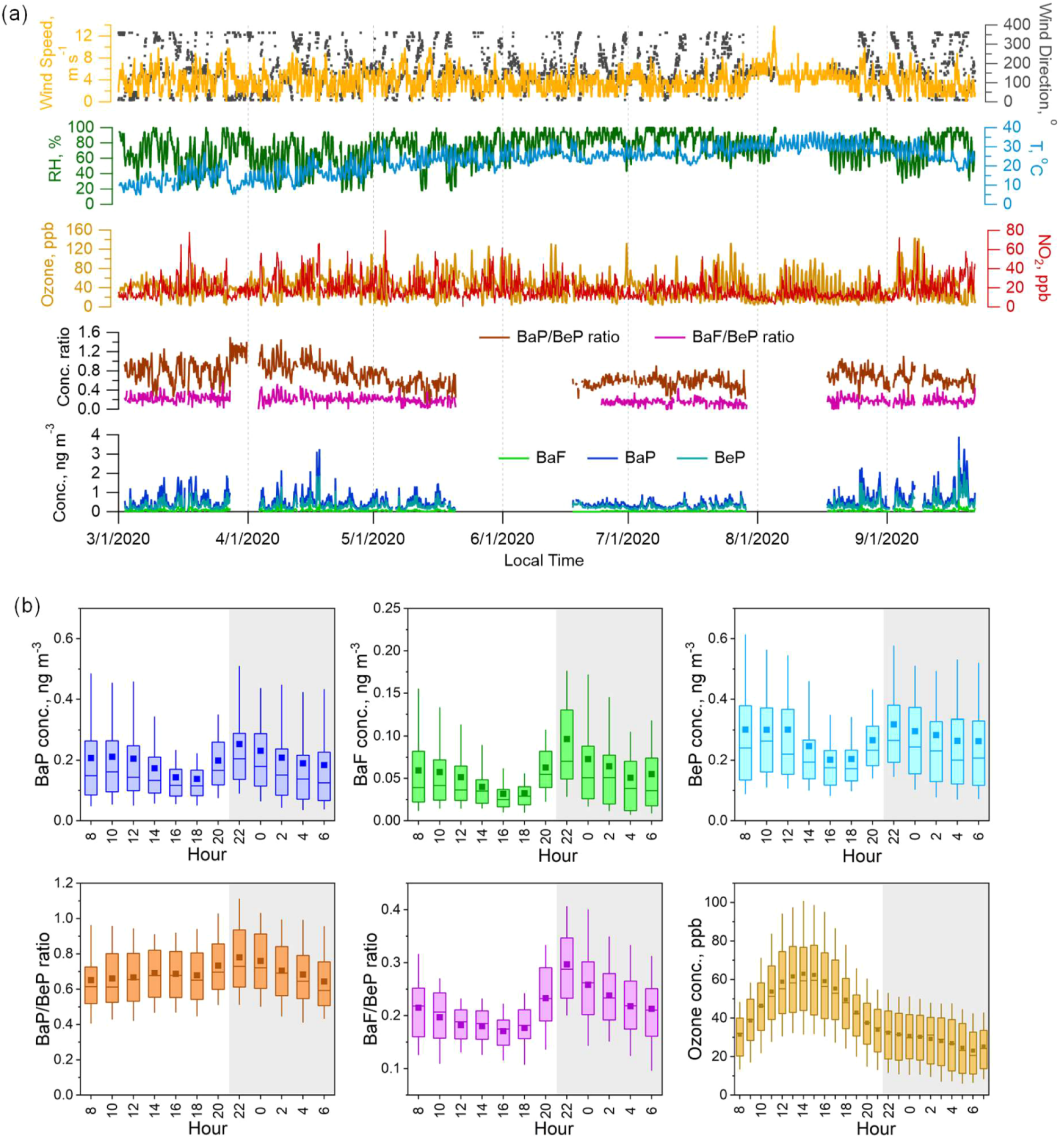
(a) Temporal variations of meteorological conditions, ozone and NO_2_, BaP/BeP and BaF/BeP ratios, and concentrations of BaP, BaF, and BeP; (b) box plot of diurnal variations of BaP, BaF, and BeP concentrations, BaP/BeP and BaF/BeP ratios, and ozone concentrations across the campaign period from March 1 to September 22, 2020, in Shanghai (squares and solid lines correspond to mean and median values, respectively; box indicates the 25th and 75th percentiles, and whiskers are the 10th and 90th percentiles). Light gray shaded area highlights the nighttime hours studied in this work (i.e., 22:00–06:00).

Figure 2b shows the average diurnal patterns of the three PAHs. A minor daytime peak at 10:00 and a pronounced nighttime peak at 22:00 were observed. Decreasing trends in BaP, BaF, and BeP concentrations were observed during two periods: the afternoon (12:00–18:00) and the nighttime (22:00–06:00). The diurnal variation in air concentrations of three PAHs was a combined effect of source emissions, chemical oxidation, and atmospheric dispersion due to planetary boundary layer height variations. In contrast, the diurnal variation of the BaP/BeP and BaF/BeP ratios was largely independent of dilution and emission effects, making it a more robust indicator of chemical oxidation processes. The diurnal variation of BaP/BeP and BaF/BeP ratios peaked at 22:00 and exhibited a clear decreasing trend throughout the nighttime window (22:00–06:00) (Figure 2b). The decreasing trend of BaF/BeP was more pronounced, consistent with BaF’s higher chemical reactivity. In contrast, the ratios during the daytime hours showed a less prominent decreasing trend. Previous laboratory studies have estimated the relative chemical reactivity of BaP toward ozone to be 1.6–500 times higher than that of BeP, depending on the aerosol matrix.^8,34–36^ The relative reactivity toward nitrating reactants is reported to be even greater (>1000).^17^ In contrast, the relative reactivity of PAHs toward ·OH oxidation is largely independent of chemical structure.^31^ This established reactivity hierarchy provides a mechanistic framework for understanding our diurnal observations. The pronounced nighttime decrease in the BaP/BeP and BaF/BeP ratios is attributed to the faster degradation of BaP and BaF by ozone or nitrating oxidants; in contrast, the stable daytime ratio reflects the similar reactivity of PAH species toward OH oxidation, which dominates daytime chemistry.

### 3.2 Calculation of the Decay Rates of BaP and BaF

Day-by-day first-order decay rate constants (*k*) for BaP and BaF were calculated by deploying the nighttime concentration ratio data during 22:00–06:00, using BeP as a reference. The decay rates represent mean values over the 8-h window, incorporating the effects of varying temperature and ozone concentrations. Of the 147 measurement days, 88 were successfully fitted by the exponential decay function (eq 1), suggesting that the reaction followed pseudo-first-order kinetics for these days. The uncertainties associated with the exponential fitting were generally low, with an average one-standard-deviation error of 23 ± 20% (range: 3.5–71%) and 17 ± 11% (range: 2.8–44%) for BaP and BaF, respectively. Two distinct scenarios were identified based on the fitting parameter A_0_, which represents the unreacted portion at the end of the night: Case#1 (A_0_ > 0), where a significant fraction of BaP (BaF) remained unreacted, and Case#2 (A_0_ = 0), where no residual BaP (BaF) remained. Representative fitting examples in both cases for BaP are shown in Figure 3. The decay fitting examples for BaF are shown in Figure S12. The time series of the derived decay rates and unreacted fractions across the entire campaign are summarized in Figure S13 and Table 1.

**Figure 3:**
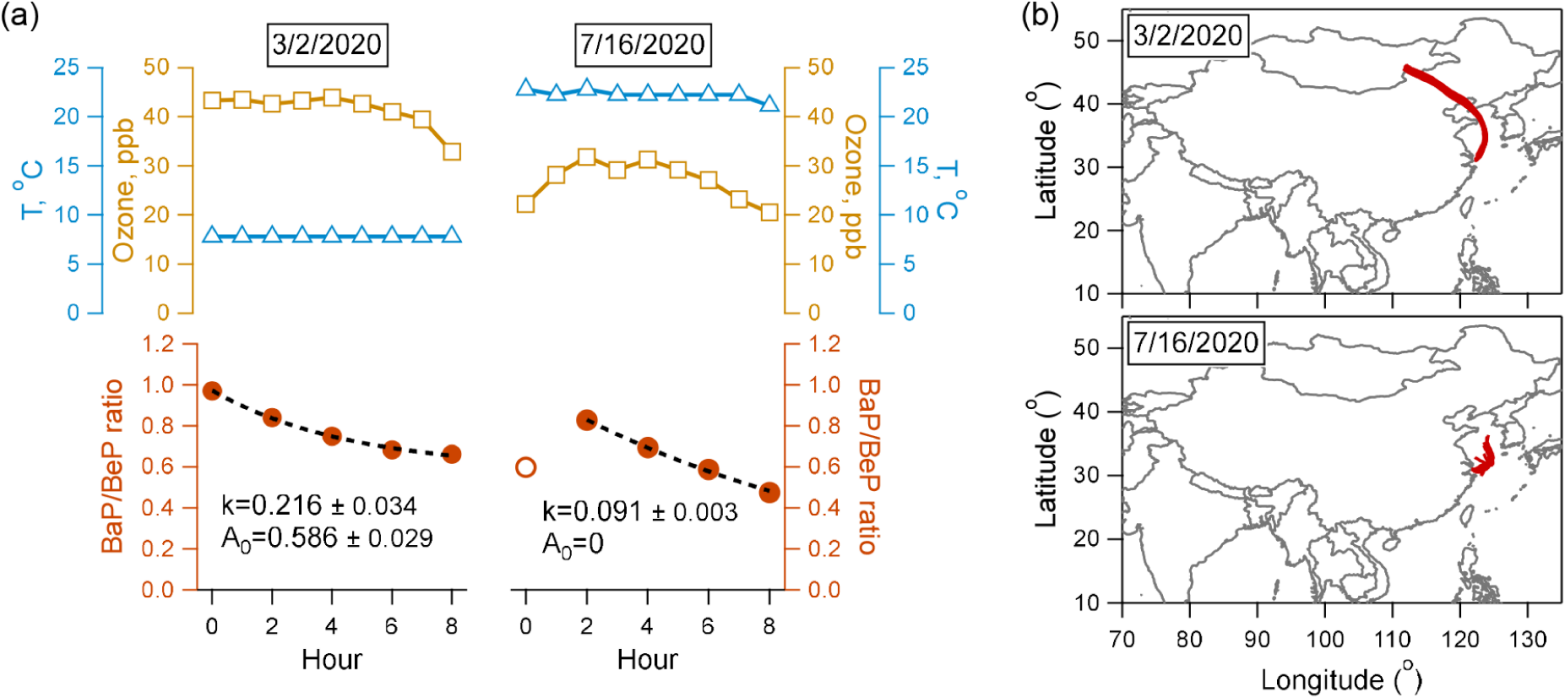
(a) Examples of the day-by-day fitting (±1 standard deviation error) of BaP normalized by BeP on two selected days (March 2, 2020, for Case#1 and July 16, 2020, for Case#2). The black dashed line shows the fitted exponential decay curve. (b) 72-h backward trajectories arriving at the sampling site at an elevation of 500 m for the 2 days.

**Table 1:** Statistical Summary of the Decay Kinetics of BaP and the Environmental Conditions under Different Temperature Regimes and across the Entire Campaign

		TR_1_ (5–16 °C)		TR_2_ (16–25 °C)		TR_3_ (25–30 °C)		Whole period	
		Avg.	Range	Avg.	Range	Avg.	Range	Avg.	Range
BaP	k (h^–1^)	0.30 ± 0.17	0.08–0.63	0.16 ± 0.11	0.03–0.48	0.11 ± 0.08	0.03–0.38	0.21 ± 0.15	0.03–0.63
	A_0_	0.55 ± 0.26	0–1.08	0.37 ± 0.19	0–0.75	0.29 ± 0.20	0–0.77	0.43 ± 0.25	0–1.08
	A_0_ fraction	53 ± 23%	0–83%	53 ± 27%	0–87%	38 ± 25%	0–81%	50 ± 25%	0–87%
	*E*_a_ (kJ mol^–1^)	125	/	118	/	204	/	/	/
	Lifetime (h)	5.2 ± 3.3	1.6–12.6	9.3 ± 4.8	2.8–29.7	13.1 ± 7.0	2.6–31.0	8.4 ± 6.0	1.6–31.0
	Initial BaP level	1.03 ± 0.20	0.59–1.46	0.73 ± 0.16	0.50–1.11	0.74 ± 0.13	0.38–0.95	0.76 ± 0.14	0.38–1.46
BaF	k (h^–1^)	0.35 ± 0.18	0.03–0.75	0.29 ± 0.15	0.07–0.56	0.23 ± 0.16	0.06–0.62	0.21 ± 0.15	0.09–0.48
	A_0_	0.16 ± 0.08	0–0.38	0.11 ± 0.08	0–0.24	0.11 ± 0.07	0–0.23	0.13 ± 0.08	0–0.38
	A_0_ fraction	49 ± 24%	0–81%	42 ± 28%	0–89%	44 ± 26%	0–84%	44 ± 25%	0–89%
	*E*_a_ (kJ mol^–1^)	68	/	/	/	163	/	/	/
	Lifetime (h)	4.2 ± 4.4	1.3–26.6	4.9 ± 3.4	1.8–14.7	6.4 ± 4.0	1.6–18.1	5.1 ± 4.0	1.3–26.6
	Initial BaF level	1.03 ± 0.20	0.59–1.46	0.73 ± 0.16	0.50–1.11	0.74 ± 0.13	0.38–0.95	0.26 ± 0.10	ND^a^–0.58
	O_3_ (ppb)	35.6 ± 11.8	4.3–57.6	30.5 ± 10.3	8.9–50.4	15.6 ± 7.10	7.5–30.8	28.2 ± 13.4	3.5–57.6
	NO (ppb)	1.57 ± 1.67	0.49–9.14	1.83 ± 2.47	0.43–14.5	2.13 ± 0.80	0.76–3.59	2.38 ± 4.69	0.22–49.0
	NO_2_ (ppb)	20.2 ± 11.2	7.17–52.1	18.7 ± 8.82	8.53–42.5	15.0 ± 5.12	9.12–28.0	18.2 ± 9.64	3.78–61.0
	T (°C)	11.5 ± 2.8	5.9–15.7	22.2 ± 2.16	16.8–25	27.4 ± 1.19	25.4–30	20.8 ± 6.6	5.9–30
	RH (%)	71.5 ± 12.6%	46–95%	84.7 ± 11.5%	58–98%	86.2 ± 8.7%	65–99%	76.6 ± 22.6%	40–100%

a not detected.

In the first example from Case#1 (March 2, 2020, Figure 3a left panel), the BaP/BeP ratio exhibited a progressively slower decay rate, decreasing by 13% in the first two h but only 3% in the final 2 h. The fitted average decay rate over the studied time window was *k* = 0.22 h^–1^, with a substantial unreacted portion A_0_ = 0.59, accounting for 60% of the initial BaP level. This occurred under conditions of an average ozone concentration of 41 ppb and average ambient temperature of 22 °C. The majority of ambient data (79 out of 88 days) belonged to this case. The residual term A_0_ averaged at 0.49 ± 0.20, ranging 0.13–1.08. The unreacted BaP accounted for 57 ± 18% (22–87%) of the initial BaP level. This is consistent with previous studies reporting unreacted fractions of 20–70% due to the shielding of BaP from oxidation by coatings of viscous organic aerosols within the aerosol matrix.^10,37^ In the second example from Case#2 (July 16, 2020, Figure 3a right panel), the BaP/BeP ratio showed a consistent and linear decay at a rate of 11–13% every 2 h. The fitted average decay rate was *k* = 0.09 h^–1^, with little unreacted fraction (A_0_ = 0), indicating the near-depletion of BaP under prolonged similar ambient conditions. Average ozone concentration on this day was 27 ppb, and average ambient temperature was 7.8 °C. This scenario was less frequent, observed only on 9 days for BaP and 7 days for BaF. The occurrence dates differed for the two species, possibly due to their different mixing states in the aerosols.

The two decay scenarios observed in this study are consistent with those of previous laboratory studies. Perraudin et al.^8^ also reported two distinct decay patterns for the ozonolysis reaction of particulate PAHs, where reactions on graphite consistently reached a “plateau”, while reactions on silica particles proceeded to completion. The dissimilar kinetic behaviors of the decay of BaP and BaF on different days may arise from differences in ambient conditions and aerosol properties. Backward air mass trajectory analysis suggested that days with complete reaction (case 2) were associated with significantly shorter trajectory lengths (Figure 3b and S14–S15), indicating an influence of fresh, locally emitted aerosols. This is further confirmed by concomitant chemical signatures of lower ozone concentrations, elevated NO levels, and higher NO/NO_2_ ratios under this case (Figure S16), characteristic of a fresh urban pollution plume.

We also calculated the decay rates of BaP using BbF as a reference, and the results are comparable to those using BeP as a reference (Figure S17), with a relative difference of 14% for the decay rates and 21% for the unreacted fraction A_0_. The comparison further supported the robustness of this method. On the other hand, decay rates of BaF were higher than those of BaP, in accordance with their relative chemical reactivities (Figure S18). Moderate correlations were observed between the two PAHs for both decay rates and unreacted fractions, suggesting similar major oxidation pathways. Thus, the decay rates of BaP are further discussed in the following analysis.

### 3.3 Factors Influencing the Decreasing Rates of BaP

To elucidate the decay mechanisms of BaP under ambient conditions, we examined the dependence of the obtained first-order rate constant (*k*) on key environmental parameters, including the gas-phase oxidant concentration, initial BaP concentration, and temperature. As measured concentrations of two reactants (i.e., oxidant and BaP) are bulk concentrations rather than the concentrations available on the aerosol surface for reaction, the correlation analysis can provide insight into the influence of diffusion processes and the aerosol matrix effect. Wang et al.^38^ observed distinct kinetic behaviors for oleic acid decay under different temperature ranges. The ambient kinetic data of BaP showed analogous patterns. Thus, to minimize the confounding influence of temperature variations, the data are stratified into three temperature regimes for subsequent analysis: TR_1_ (5–16 °C), TR_2_ (16–25 °C), and TR_3_ (25–30 °C).

Figure 4 plots the dependence of the decay rates on average nighttime oxidants (O_3_ and NO_3_*) and the initial BaP concentration. The NO_3_*, calculated as [O_3_] × [NO_2_], was used as a surrogate to represent NO_3_ radical concentrations. A positive correlation with ozone was observed across three temperature regimes, while the decay rates showed positive correlations with only NO_3_* under TR_2–3_. The results indicated that the heterogeneous reaction of BaP was ozone-limited under TR_1_, while both O_3_ and NO_3_ were important under TR_2–3_. Previous laboratory studies have reported both linear and nonlinear Langmuir–Hinshelwood behavior of BaP decay upon ozone oxidation (Table S2). Our ambient data exhibit a moderate linear correlation. This is likely attributable to the relatively low ambient ozone concentrations (<60 ppb), which would fall within the linear range of Langmuir–Hinshelwood behavior or synergistic effects of different oxidants. In contrast, the dependence on the initial BaP concentration varied with temperature. Under TR_1_, decay rates exhibited a negative correlation (R_p_: −0.65), while a positive correlation was observed under TR_2_ (R_p_: 0.32). No clear relationship was noticed under TR_3_. The negative correlation in TR_1_ is consistent with laboratory studies showing that higher PAH loadings on solid particles enhance surface/bulk shielding effects, thereby reducing the reaction rate.^8,12,13^ The positive correlation under TR_2_ suggests a different aerosol morphology, where increased BaP concentrations may enhance its diffusion from the particle bulk to the surface, facilitating the reaction. Thus, under TR_1–2_, the decay rate is influenced by both the oxidant and initial BaP concentration, albeit through different mechanisms. Under TR_3_, however, the reaction rate became independent of the initial BaP loadings.

**Figure 4:**
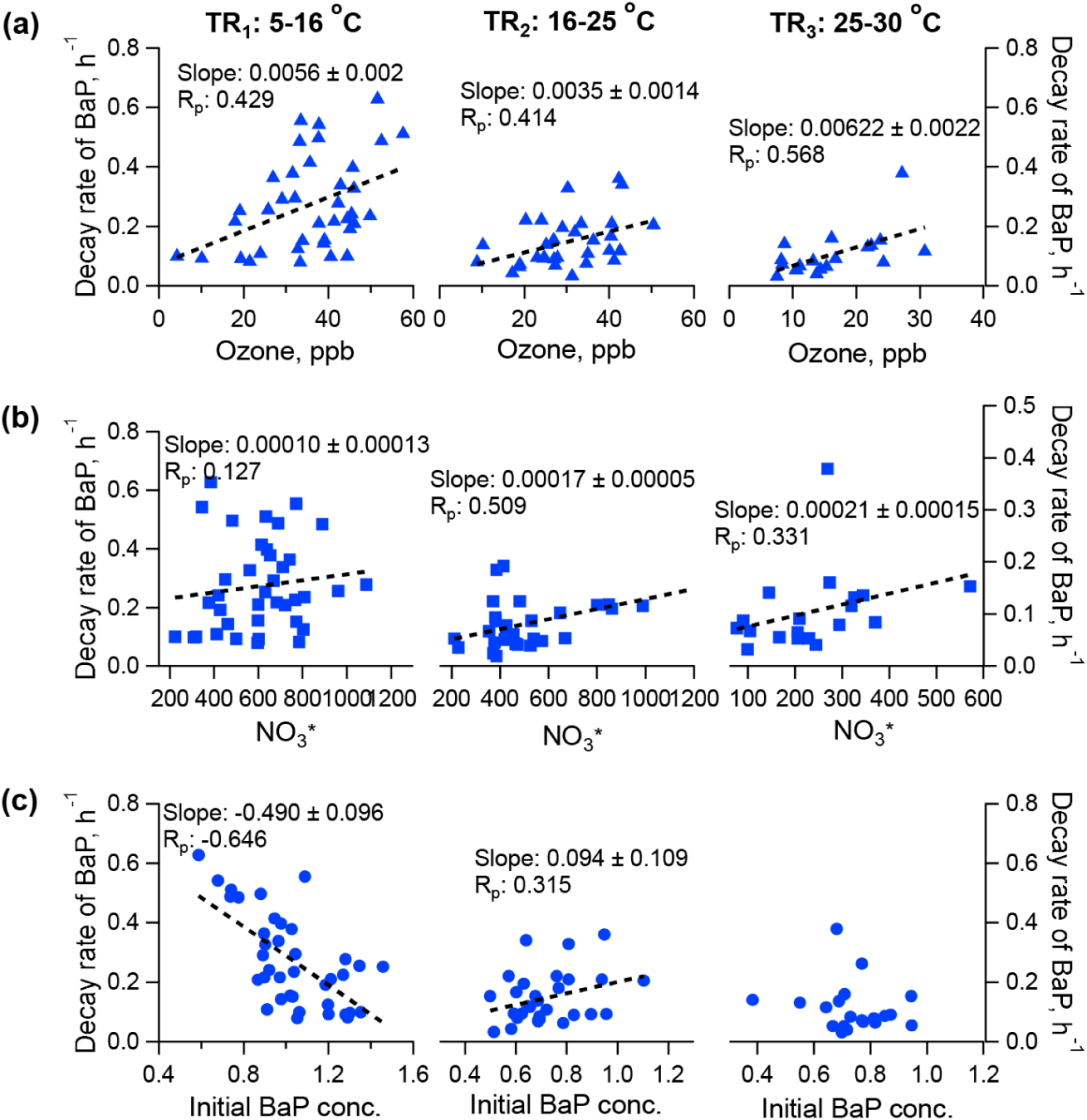
Correlations of the estimated effective decay rate of BaP with the (a) average nighttime O_3_ concentration, (b) NO_3_* concentration, and (c) initial BaP concentration. Dash line is the linear fitting.

The temperature dependence of BaP decay is further illustrated by the Arrhenius plot, which displays the natural logarithm of the decay rates against the inverse of the temperature (Figure 5). The results for BaF are shown in Figure S19. The ambient data were segregated into different clusters corresponding to different temperature regimes (TR_1–3_). Linear correlations were observed with an R_p_ of 0.48–0.67 under TR_1–3_. The effective activation energy (*E*_a_) for BaP decay, derived from the slopes of the linear fits (−*E*_a_/R), was 125, 118, and 204 kJ mol^–1^ for TR_1_, TR_2_, and TR_3_, respectively. The comparable *E*_a_ values under TR_1–2_ are slightly higher than the value reported for the reaction between BaP and reactive oxygen intermediates (ROIs) on soot particles (∼80 kJ mol^–1^).^39^ In contrast, the significantly higher *E*_a_ observed under TR_3_ suggested that the reaction was more sensitive to temperature variations. This elevated value suggests that the oxidation reaction was substantially hindered under the high-temperature, high-RH, and low-ozone conditions of TR_3_. The results are in agreement with findings for oleic acid decay under ambient conditions.^38^

**Figure 5:**
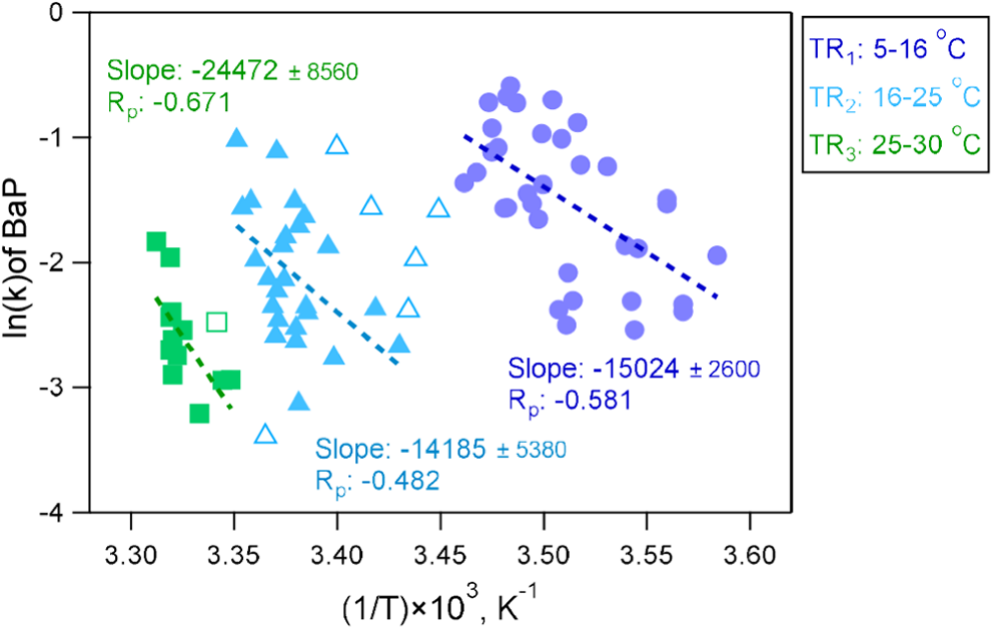
Arrhenius plot of the decay rates (k) of BaP with ambient temperature. Data are shown in different colors, indicating different temperature ranges. Solid markers represent the data used for the linear fitting of ln­(k) vs 1/T for each temperature range. The calculated slope is positively associated with the effective activation energy for each scenario.

### 3.4 Factors Influencing the Unreacted BaP

The presence of an unreacted fraction prolongs the atmospheric lifetime of PAHs, facilitating their long-range transport to remote places. This is supported by our calculations, which identified unreacted BaP and BaF (A_0_) primarily in aged ambient aerosols that had undergone substantial atmospheric transport. As A_0_ denotes the normalized concentration of BaP relative to BeP upon completion of the reaction, the fraction of unreacted BaP was subsequently calculated by dividing A_0_ by the initial normalized BaP concentration at 22:00. We then examined the relationship between the fraction of unreacted BaP and key environmental factors. Perraudin et al.^8^ reported that the unreacted PAH correlated with 1/k (or lifetime) of PAHs and depended on the nature of the particles under controlled conditions. Our study under complex ambient conditions revealed no such clear correlation between the fraction of unreacted BaP and 1/k. This discrepancy is likely attributable to the concurrent influence of multiple variable environmental parameters. However, we found clear correlations between the fraction of unreacted BaP and OC and RH, as shown in Figure 6. The fraction of unreacted BaP was positively correlated with the OC mass under TR_1_ (R_p_: 0.53), indicating that higher OC loadings were associated with a larger unreacted BaP fraction. This correlation diminished under TR_2–3_. Laboratory studies suggested that solid organic coatings substantially suppress the PAH oxidation, while liquid organic coatings did not significantly affect the kinetics.^9,36^ The results may also indicate the transition of the particle phase state, from a solid and semisolid state in TR_1_ to a liquid state in TR_3_. A_0_ exhibited a negative correlation with ambient RH under TR_1–3_ (R_p_: −0.31 to 0.42). The aerosol viscosity is closely associated with ambient temperature and RH; increased RH reduces aerosol viscosity, enhancing the diffusion of BaP molecules to the particle surface for oxidation and thereby resulting in a smaller unreacted fraction.

**Figure 6:**
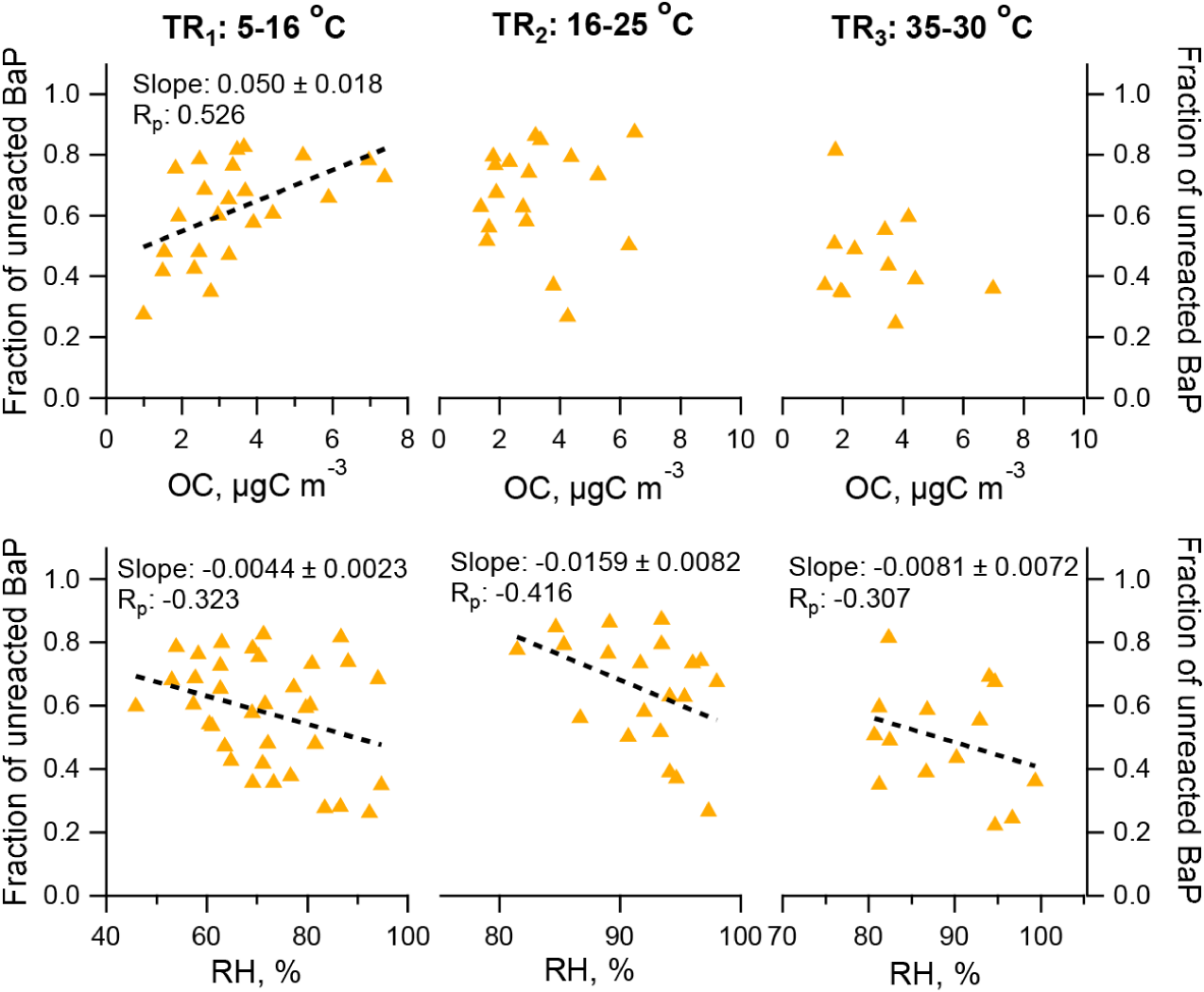
Correlations of the fraction of unreacted BaP with the average OC concentration and ambient RH. Data are presented by different temperature ranges (TR_1–3_). Dash line is the linear fitting line.

### 3.5 Lifetime and Atmospheric Implication

The nighttime atmospheric lifetime of BaP and BaF was estimated using the obtained effective first-order rate constant. Average lifetimes of BaP and BaF across the campaign period were 8.4 and 5.1 h, respectively, under an ozone level of 28 ppb. The lifetime of BaF was shorter than BaP, especially under TR_3_ (Table 1). Day-by-day nighttime lifetimes of BaP in this study are shown in Figure 7, together with estimates from previous laboratory-based studies. The BaP lifetime in this study was negatively associated with ambient temperature and ozone level within each temperature regime. However, lifetimes under high-temperature conditions could not be directly extrapolated from low-temperature data, indicating a nonlinear and complex dependence of BaP heterogeneous oxidation on environmental conditions. This pattern is consistent with observations for ambient oleic acid, which also showed prolonged lifetime at higher temperatures in summer.^38^ Lifetimes of BaP from previous laboratory studies were estimated assuming an ozone level of 30 ppb based on the parameters summarized in Table S2. Given that most laboratory experiments were conducted at room temperature (∼25 °C) and dry conditions (RH < 5%), we focus our comparison on the TR_2_ regime (16–25 °C). The ambient lifetime of BaP under TR_2_ (9.3 h) in this study aligned closely with values reported for BaP adsorbed on ammonium sulfate (AS) coated with thick secondary organic aerosols (SOA) (7.5 h),^36^ solid azelaic acid particles (6.5 h),^40^ and graphite (7.1 h).^8^ In contrast, it was substantially higher than the results on bare AS particles,^9^ soot particles,^13^ and liquid octanol films.^41^ The results reveal that the heterogeneous oxidation kinetics of particulate PAHs is governed by a complex interplay between ambient conditions and the physicochemical properties of the aerosol matrix. This study establishes that high-time resolution measurements of select ratios of PAH pairs can be used to quantify their decay kinetics, providing valuable kinetic data pertaining to real-world conditions. This approach is broadly applicable to other reactive PAHs, enabling the acquisition of kinetic data critical for modeling atmospheric processes.

**Figure 7:**
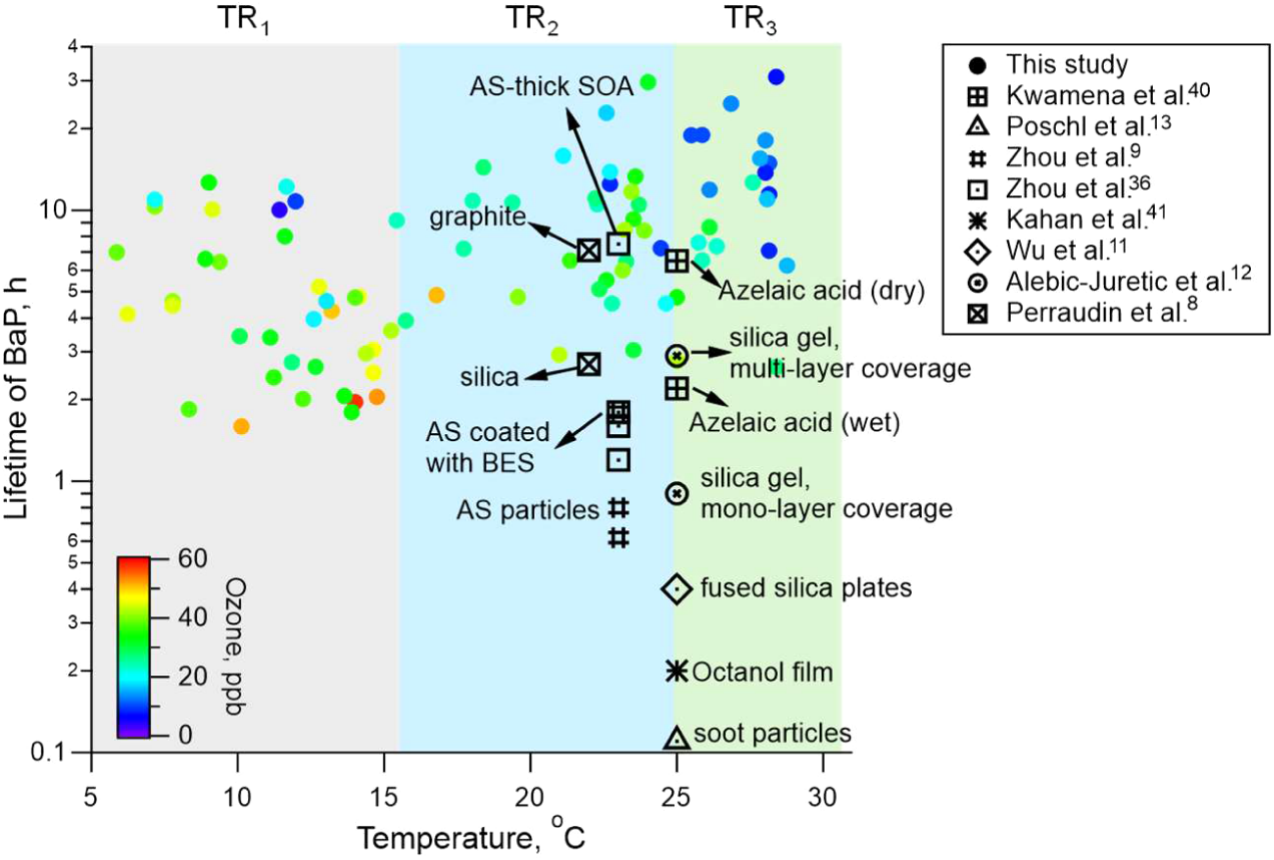
Day-by-day nighttime lifetimes of BaP obtained in this study and comparison with estimates from previous laboratory studies assuming an ozone level of 30 ppb. Ambient data in this study are color-coded by ozone concentrations.

## Supplementary Material



## Notes

Quality assurance details of online measurements of PAHs by TAG; statistical summary, temporal variation, and correlation analysis of measured PAHs; derivation details of decay rates for BaP using the relative rate constant approach; source apportionment analysis of BaP, BaF, and BeP; examples of the exponential decay fitting for BaF; temporal variations and comparisons of decay rates of BaF and BaP; comparison of BaP decay rates derived using BeP as reference versus those using BbF as reference; backward trajectories for days with no BaP (BaF) left during the decay fitting; Arrhenius plot of decay rates of BaF with temperature; summary of decay rates of BaP from previous laboratory studies (PDF)
