# Supplementary material for: Day-by-Day Ambient Determination of Nighttime Heterogeneous Oxidation of Particle-Phase Benzo[a]pyrene in an Urban Environment
Source: Environ Sci Technol. 2026 Apr 7;60(15):11621–30. doi: 10.1021/acs.est.6c02669 (PMC13104171; doi:10.1021/acs.est.6c02669)
Supplement: Supplementary file 1 [file es6c02669_si_001.pdf]

Supporting Information for

Day-by-Day Ambient Determination of Nighttime Heterogeneous Oxidation of Particle-phase Benzo[a]pyrene in an Urban Environment

Qiongqiong Wang[1,2], Zongjun Li[1], Zhongliang Huang[1], Shuhui Zhu[2,*], Jie Li[3], Liping Qiao[2], Min Zhou[2], Dandan Huang[2], Hongli Wang[2], Qingyan Fu[2], Huan Yu[1], Jian Zhen Yu[4,*]

[1] Department of Atmospheric Science, School of Environmental Studies, China University of Geosciences, Wuhan 430074, China.

[2] Laboratory of Formation and Prevention of Urban Air Pollution Complex, Ministry of Ecology and Environment, Shanghai Academy of Environmental Sciences, Shanghai 200233, China.

[3] Institute of Molecular Aggregation Science, Tianjin University, Tianjin 300072, China.

[4] Department of Chemistry, Hong Kong University of Science and Technology, Hong Kong 999077, China.

[*]Corresponding author: Shuhui Zhu (zhush@saes.sh.cn) and Jian Zhen Yu (chjianyu@ust.hk)

This document contains 19 figures, 2 tables, and 3 text sections as supplementary material.

Text S1. Quality Assurance of PAH Quantification

Bihourly concentrations of ambient PAHs were measured using TAG from March 1 to September 22, 2020, at the Shanghai Academy of Environmental Sciences (SAES, 31.17°N, 121.43°E), a typical urban site in southwestern Shanghai, China. An hourly sample at every even hour was collected, resulting in a total of 12 samples per day. A total of 1785 ambient samples were collected. Figure S1 shows the temporal variations of the sample flows during the entire campaign period. Figure S2 shows the temporal variations of peak areas of the four PAH internal standards (ISs). The entire campaign period can be divided into four sub-periods (P1-P4), during which instrument maintenance was performed. Specifically, measurement data were not available during the following periods: 18:00 on March 31 to 10:00 on April 3, 18:00 on May 20 to 14:00 on June 17, and 10:00 on July 29 to 14:00 on August 17. The peak areas of four ISs (i.e., phenanthrene-d_{10}, chrysene-d_{12}, perylene-d_{12}, and benzo[ghi]perylene-d_{12}) within each sub-period showed a gradual decreasing trend, reflecting normal degradation of instrument performance as sampling proceeded. The coefficient of variation (CV) was 16-60%, 23-66%, 26-66% and 24-71% for phenanthrene-d_{10}, chrysene-d_{12}, perylene-d_{12}, and benzo[ghi]perylene-d_{12}, respectively. Peak areas among the four ISs showed good correlations with each other (R_p: 0.98-0.99) (Figure S3). Quantification of the individual PAH species was achieved using external authentic standards and four ISs. One calibration curve was built for each sub-period and four calibration curves in total were available during the entire campaign period. Pearson correlation coefficients (R_p) of the calibration curves for PAH species were >0.99.

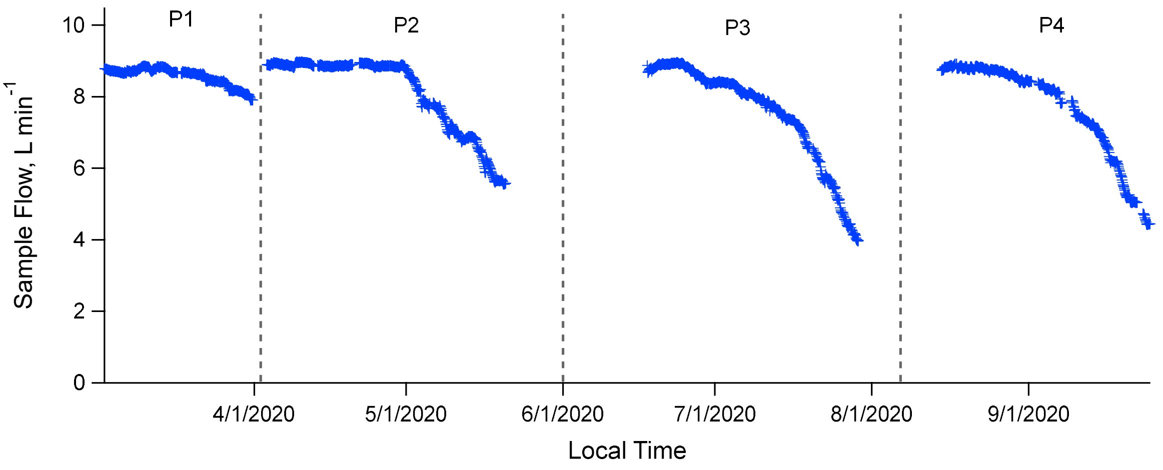

Figure S1. Time series of the sample flow across the entire campaign period.

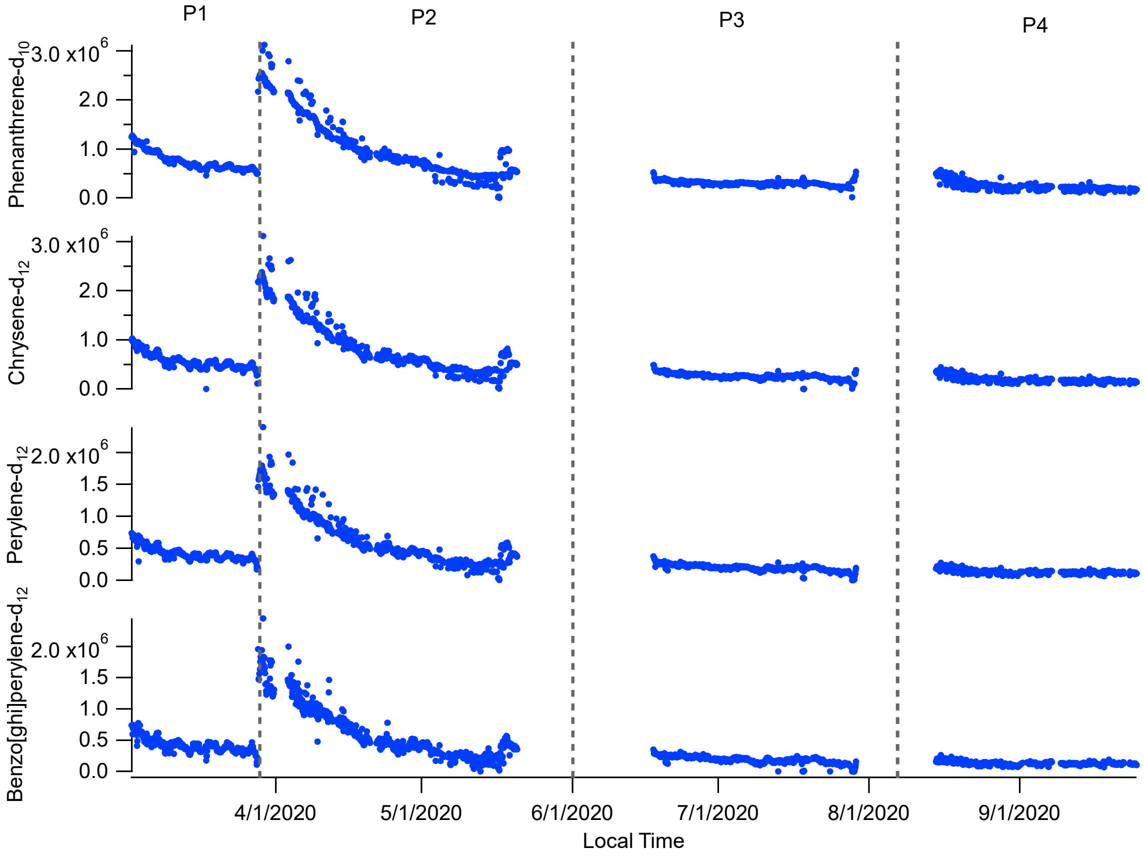

Figure S2. Temporal variations of the peak areas of four PAH ISs during the campaign period.

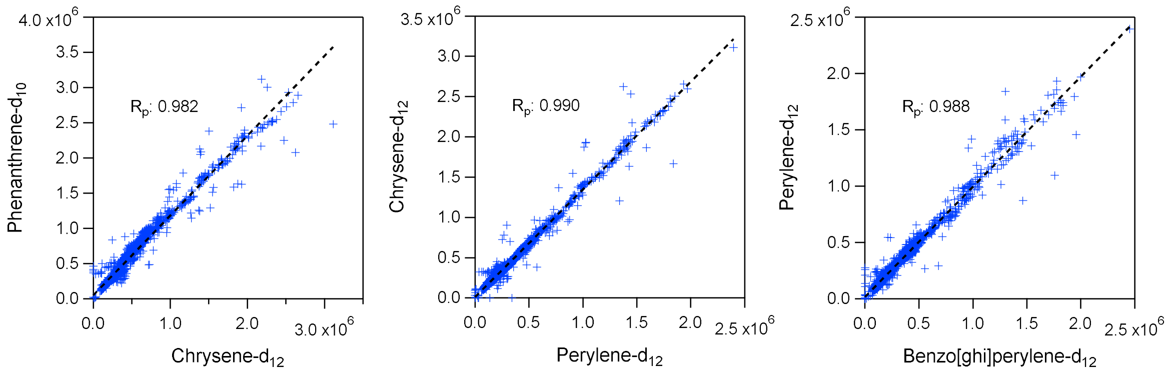

Figure S3. Correlation of the peak areas among four PAH ISs.

Text S2. Derivation of the Decay Rate for BaP Using the Relative Rate Constant Approach

The detailed derivation of the ambient decay rates for aerosol organic species can be found in our previous work.[1,2] Here, we briefly present main principles for deducing the equations for particle-borne BaP. The rate expression for the concentration of BaP in the atmosphere can be written as follows:

$$\frac{\partial C_{BaP}}{\partial t} = -k_{r_{BaP}} \cdot C_{OX} \cdot C_{BaP} \cdot (1 + f_{BaP}) - k_{d_{BaP}} \cdot C_{BaP} - k_{d'_{BaP}} \cdot C_{BaP} + E_{BaP} \quad (1)$$

$$f_{BaP} = < \frac{C'_{OX} \cdot C'_{BaP}}{C_{OX} \cdot C_{BaP}} > \quad (2)$$

$k_{r_{\mathrm{BaP}}}$ is the second-order reaction rate constant of BaP, C_{BaP} is the measured concentration of particle-borne BaP, C_{OX} is the average oxidant concentration in the aerosol, $k_{d_{\mathrm{BaP}}}$ and $k_{d'_{\mathrm{BaP}}}$ are the dilution and deposition rate constants of BaP, E_{BaP} is the source emission rate of BaP, f_{BaP} is a fractional covariance term that describes spatial variations of the reactants within the particle. $f_{\mathrm{BaP}} = 0$ if either the oxidant or the reactant BaP is well mixed (i.e., gradients C'_{OX}=0 or C'_{BaP}=0). $f_{\mathrm{BaP}} > 0$ when BaP and oxidant are mainly at the particle surface. $f_{\mathrm{BaP}} < 0$ when BaP is depleted near the particle surface and the oxidant is concentrated near the particle surface.[3]

By using the normalized concentration, i.e., the concentration of BaP normalized by a reference species (BeP in this case), the interference from source emissions, atmospheric dilution and deposition can be eliminated:

$$\frac{\partial \frac{C_{\mathrm{BaP}}}{C_{\mathrm{BeP}}}}{\partial t} = -\left\{k_{r_{\mathrm{BaP}}} \cdot C_{OX} \cdot (1 + f_{\mathrm{BaP}}) - k_{r_{\mathrm{BeP}}} \cdot C_{OX} \cdot (1 + f_{\mathrm{BeP}})\right\} \cdot \frac{C_{\mathrm{BaP}}}{C_{\mathrm{BeP}}} - \left(k_{d_{\mathrm{BaP}}} - k_{d_{\mathrm{BeP}}}\right) \cdot \frac{C_{\mathrm{BaP}}}{C_{\mathrm{BeP}}} - \left(k_{d'_{\mathrm{BaP}}} - k_{d'_{\mathrm{BeP}}}\right) \cdot \frac{C_{\mathrm{BaP}}}{C_{\mathrm{BeP}}} - (E_{\mathrm{BeP}} \cdot C_{BaP} - E_{\mathrm{BaP}} \cdot C_{BeP})/{C_{BeP}}^2 \quad (3)$$

It is reasonable to assume same deposition and dilution rates for BaP and BeP, i.e., $k_{d_i \mathrm{BaP}} = k_{d_{\mathrm{BeP}}}$, $k_{d'_{\mathrm{BaP}}} = k_{d'_{\mathrm{BeP}}}$. Assuming the reaction occurs at or near the aerosol surface, the reagent concentration within the particle is well mixed initially, thus, $f_{\mathrm{BaP}} = f_{\mathrm{BeP}} = 0$:

$$\frac{\partial \frac{C_{\mathrm{BaP}}}{C_{\mathrm{BeP}}}}{\partial t} = -(k_{r_{\mathrm{BaP}}} - k_{r_{\mathrm{BeP}}}) \cdot C_{OX} \cdot \frac{C_{\mathrm{BaP}}}{C_{\mathrm{BeP}}} - (E_{\mathrm{BeP}} \cdot C_{BaP} - E_{\mathrm{BaP}} \cdot C_{BeP})/{C_{BeP}}^2 \quad (4)$$

As demonstrated in Text S3, ambient BaP and BeP exhibited strong correlations with one another and shared common sources. During the selected 8-h time window (22:00-06:00), we assume that fresh emissions were absent or negligible, i.e., $E_{Bep} \cong 0$ and $E_{BaP} \cong 0$, or the variation of the concentration ratio over time was governed primarily by chemical reactions (the first term on the right-hand side of Eq.4) rather than by emission-related processes (the second term on the right-hand side of Eq.4). For sampling days that satisfied this assumption, Eq. 5 was derived as follows:

$$\frac{\partial \frac{C_{\mathrm{BaP}}}{C_{\mathrm{BeP}}}}{\partial t} = -(k_{r_{\mathrm{BaP}}} - k_{r_{\mathrm{BeP}}}) \cdot C_{OX} \cdot \frac{C_{\mathrm{BaP}}}{C_{\mathrm{BeP}}} \quad (5)$$

From previous laboratory studies, chemical reactivity of BaP towards ozone and nitrating species is much higher than that of BeP,[4,5] i.e., $k_{r_{\mathrm{BaP}}} \gg k_{r_{\mathrm{BeP}}}$. As the studied time window was during nighttime, interference from ·OH oxidation was minimal, and the decay of BaP was mainly contributed by ozone and nitrating species. Thus, Eq. 5 can be approximated as:

$$\frac{\partial \frac{C_{\mathrm{BaP}}}{C_{\mathrm{BeP}}}}{\partial \mathrm{t}} \approx -k_{r_{\mathrm{BaP}}} \cdot C_{OX} \cdot \frac{C_{\mathrm{BaP}}}{C_{\mathrm{BeP}}} \qquad (6)$$

$$\frac{C_{\mathrm{BaP}}}{C_{\mathrm{BeP}}} = A_1 \cdot e^{-k \cdot t} + A_0 , \qquad k \approx k_{r_{BaP}} \cdot C_{ox} , \quad A_0 \geq 0 \qquad (7)$$

Here, we provide a general equation with a residual term A_0 representing the unreacted BaP during the studied time window (Eq. 7). If no BaP remains, $A_0=0$. If $A_0>0$, the reacted portion belongs to the exponential decay function. It should be noted that these assumptions are likely valid only for a subset of days within the campaign, given the inherent complexity of the real atmosphere. Variability in source emissions and the heterogeneous nature of the aerosol matrix may introduce additional uncertainties into the derived decay rates.

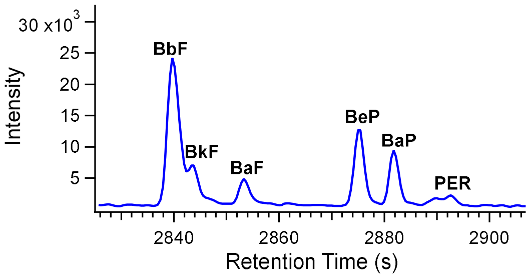

Figure S4. Typical TAG chromatogram of BaP and its five isomers from ambient samples collected in Shanghai.

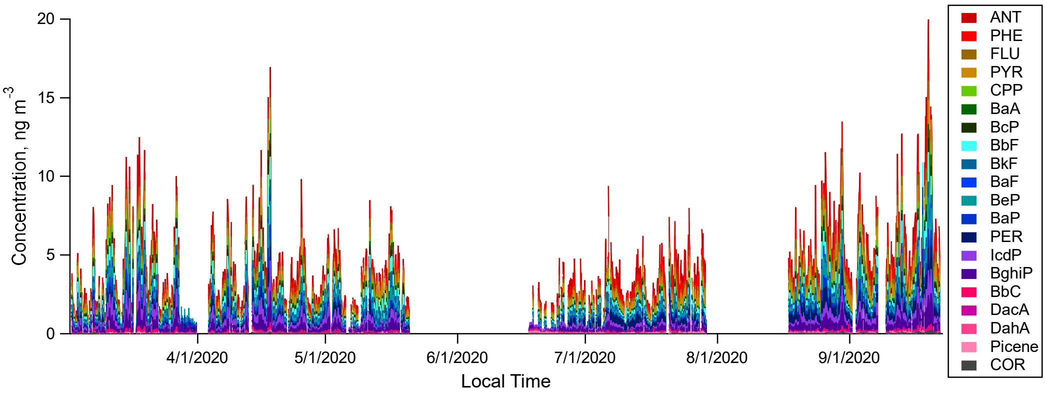

Figure S5. Temporal variations of individual PAH concentrations across the campaign period from March 1 to September 22, 2020 in Shanghai, China.

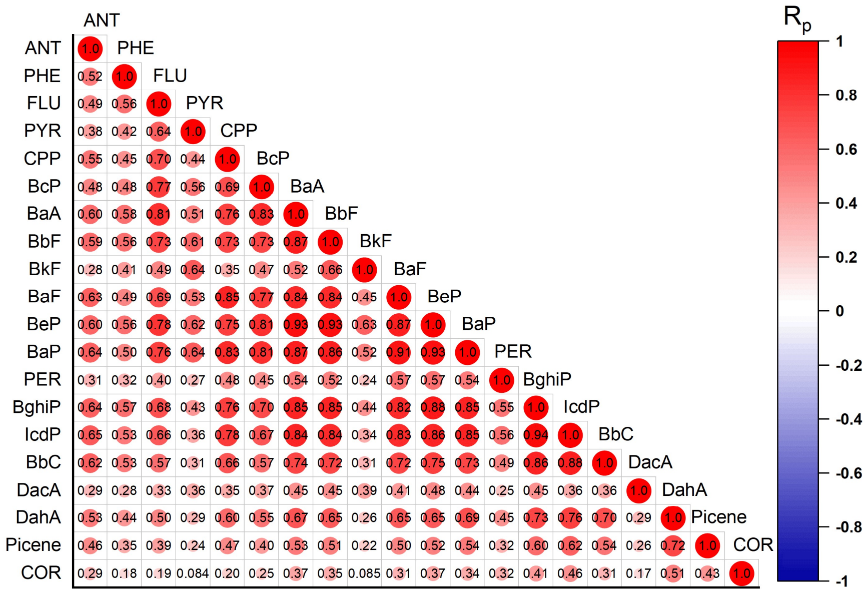

Figure S6. Pearson correlations of individual PAHs across the campaign period.

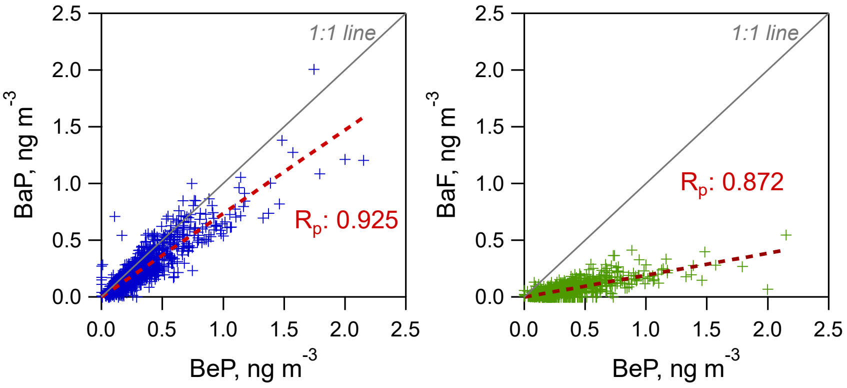

Figure S7. Correlations between BaP, BaF and BeP across the campaign period.

Text S3. Source Apportionment Analysis of BaP, BaF and BeP

To evaluate the major sources contributing to the ambient level of the three studied PAHs, we performed source apportionment analysis using the positive matrix factorization (PMF) receptor model. PMF is a bilinear factor analysis tool to resolve the source profiles and contributions, assuming chemical mass balance between the emission sources and the receptor site.[6] A comprehensive list of key organic tracers measured by TAG and inorganic tracers including ions and elements was incorporated to resolve

more detailed source information. The whole dataset was divided into three sub-periods based on the measurement continuity, i.e., March-May, June-July, and August-September, with a total data matrix of 591×26 (samples×species), 773×26, and 219×26, respectively. The input uncertainty for each species was calculated as (concentration×EF + 1/3 × MDL), where MDL is the method detection limit. For concentrations below MDL, the uncertainty was set as 5/6 × MDL.[7] The EF was set as 0.15 for organic carbon (OC), elemental carbon (EC) and elements, 0.2 for major ions, and 0.1 for organic tracers.[8,9]

Five to 14 factors were tested and the 12-factor solutions were found to be most reasonable. Figure S8 shows the factor profiles and Figure S9 shows the diurnal variations of the factor contributions to $PM_{2.5}$ for the 12-factor solutions. The results all passed the bootstrap error estimations, with >90% mapping of all bootstrap factors. Four secondary sources and eight primary sources were resolved. Source identification is based on the established marker species. Briefly, secondary sulfate and nitrate formation processes are identified by sulfate and nitrate; aromatic secondary organic aerosol (SOA), monoterpene SOA, and isoprene SOA factors are identified by the corresponding SOA tracers. Biomass burning is identified by levoglucosan and mannosan; power plant combustion is identified by Cr, Zn, Se and Pb. Two factors are related to vehicle emissions: one is vehicle exhaust, identified by hopanes and the other is tire wear, identified by Mn, Fe and Zn. The two factors had their highest contributions during morning (8:00-10:00) and afternoon rush hours (20:00-22:00), as can be seen in the diurnal variations (Figure S9). The PMF-resolved vehicle emission factors contain both gasoline and diesel emissions. Cooking emission factor is resolved by two abundant fatty acids—palmitic and stearic acids. Residual oil combustion is identified by V and Ni, and dust by Ca and Ba. BaF, BaP, and BeP stand out as a separate factor and this factor is identified as coal combustion, which is in agreement with previous studies at this observational site.[9] Detailed source identification can be found in Zhu et al.[9]

Figure S10 shows the average factor contributions to the three PAHs. Coal combustion was the major source contributing to the ambient PAH level, with contributions of 98-99% to BaF, 44-71% to BeP, and 53-77% to BaP, respectively. It shows moderate correlations with CO, suggesting a regional feature of the source (Figure S11). The sources for BaP and BeP in August-September were more diverse, with non-negligible contributions from biomass burning and vehicle emission. About 11% and 32% of BeP and 18% and 23% of BaP were from biomass burning and vehicle emission, respectively. As local biomass burning activities are prohibited in urban Shanghai, the observed biomass burning pollution

can be attributed to regional transport, a conclusion supported by its strong correlation with CO (Figure S11). The post-rush hour contribution of vehicle emission factors in the nighttime should be minimal. The source apportionment analysis provided solid evidence of the common major sources for BaF, BaP, and BeP, as well as the regional features of the main sources.

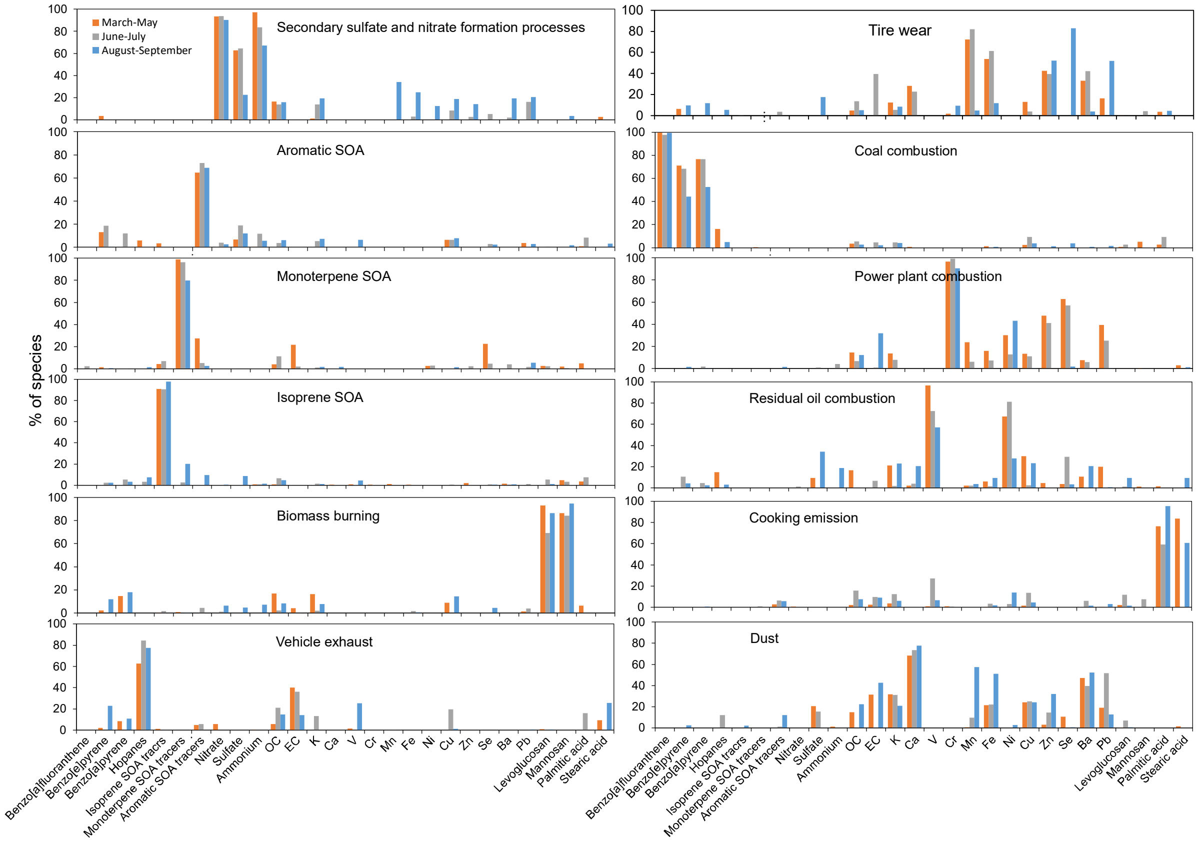

Figure S8. Explained variations (percentage of individual species in each factor) of the 12-factor solution resolved by PMF. The whole dataset was separated into three cases for PMF input (i.e., March-May, June-July, and August-September).

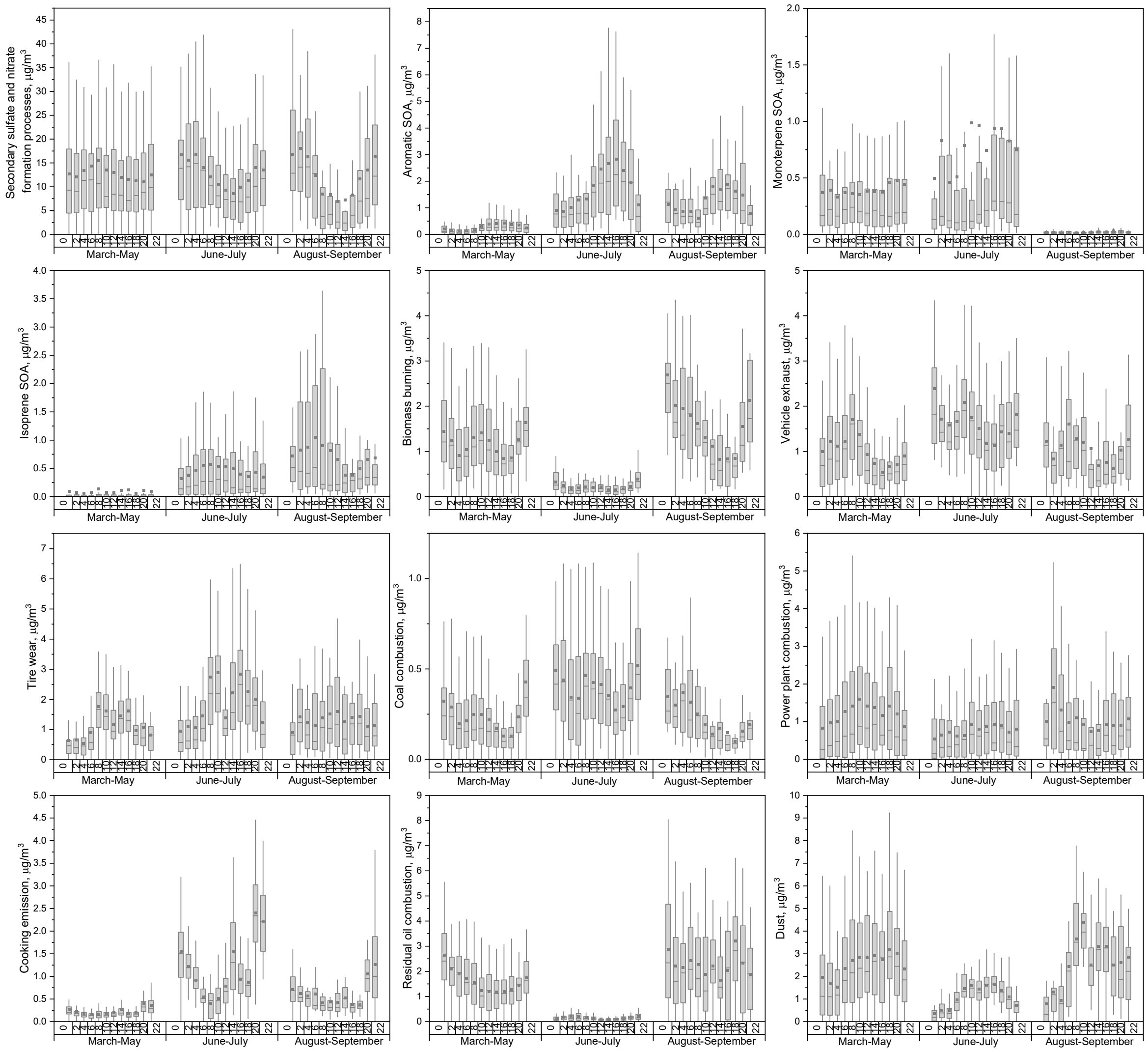

Figure S9. Diurnal variations of the source contributions to $PM_{2.5}$ from 12-factor solution resolved by PMF. The whole dataset was separated into three cases for PMF inputs (i.e., March-May, June-Julys and August-September).

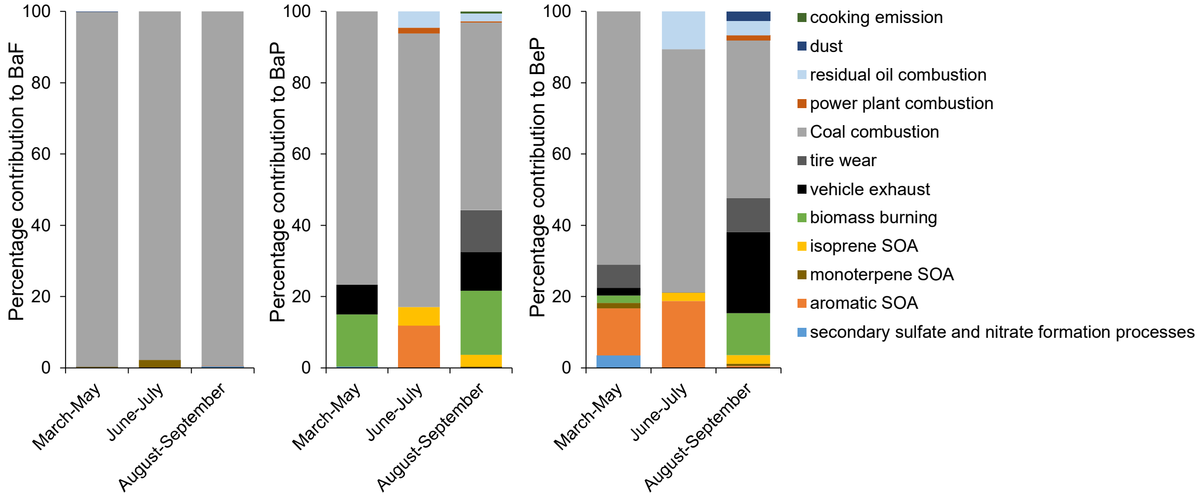

Figure S10. Percentage source contributions to BaF, BaP, and BeP from the 12-factor solution resolved by PMF.

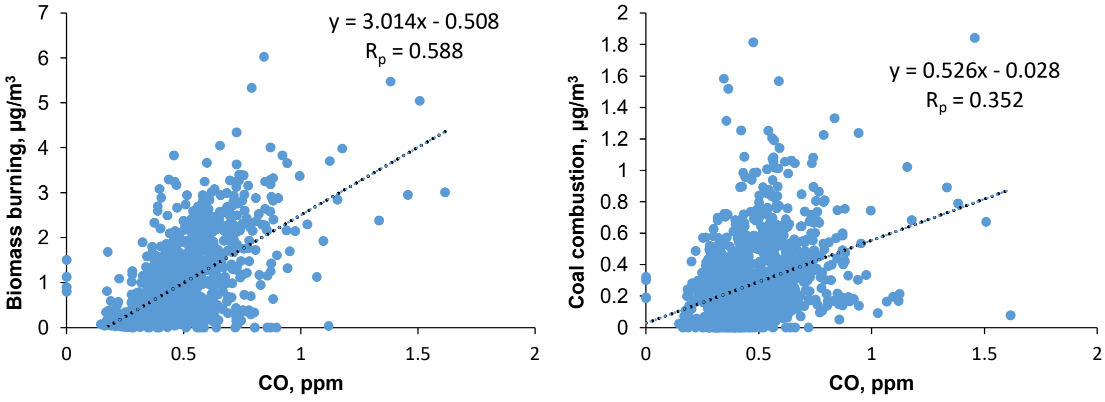

Figure S11. Correlations of source contributions from biomass burning and coal combustion with CO.

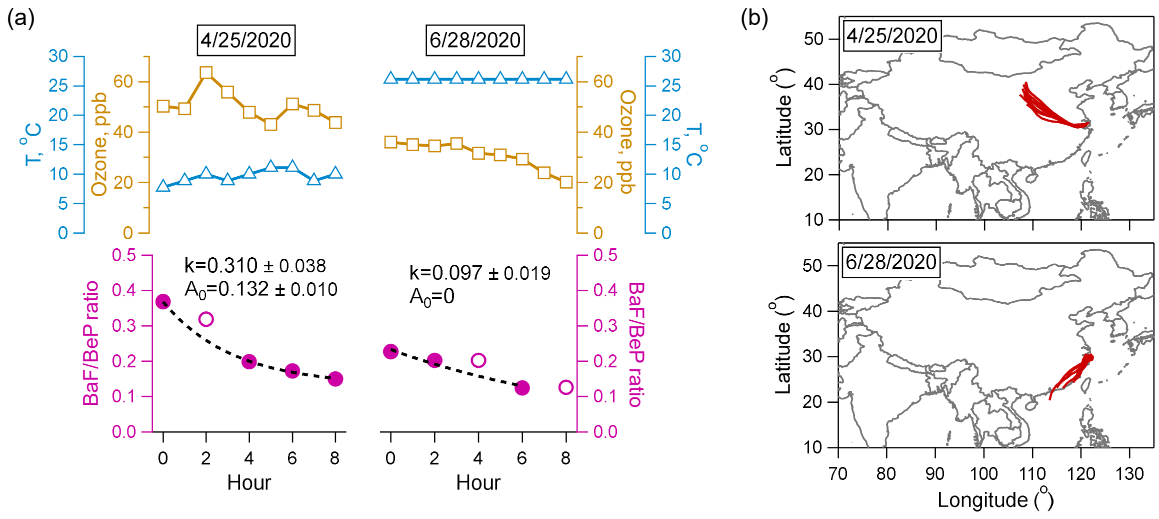

Figure S12. (a) Example of the day-by-day fitting (±1 standard deviation error) of BaF normalized by BeP on two selective days (April 25, 2020 for Case #1 and June 28, 2020 for Case#2). The dashed line is the fitting line in the exponential fitting. (b) 72-hr backward trajectory arriving at the sampling site at an elevation of 500 m for the two days.

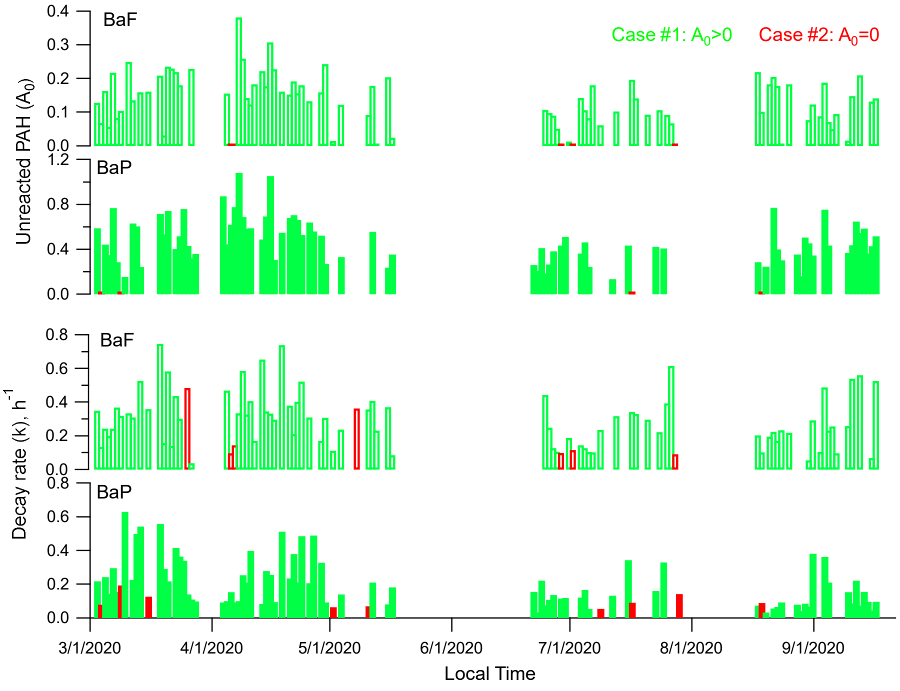

Figure S13. Time series of the calculated decay rates (k) and remaining fraction (A_0) for BaP and BaF across the campaign period from March 1 to September 22, 2020 in Shanghai, China.

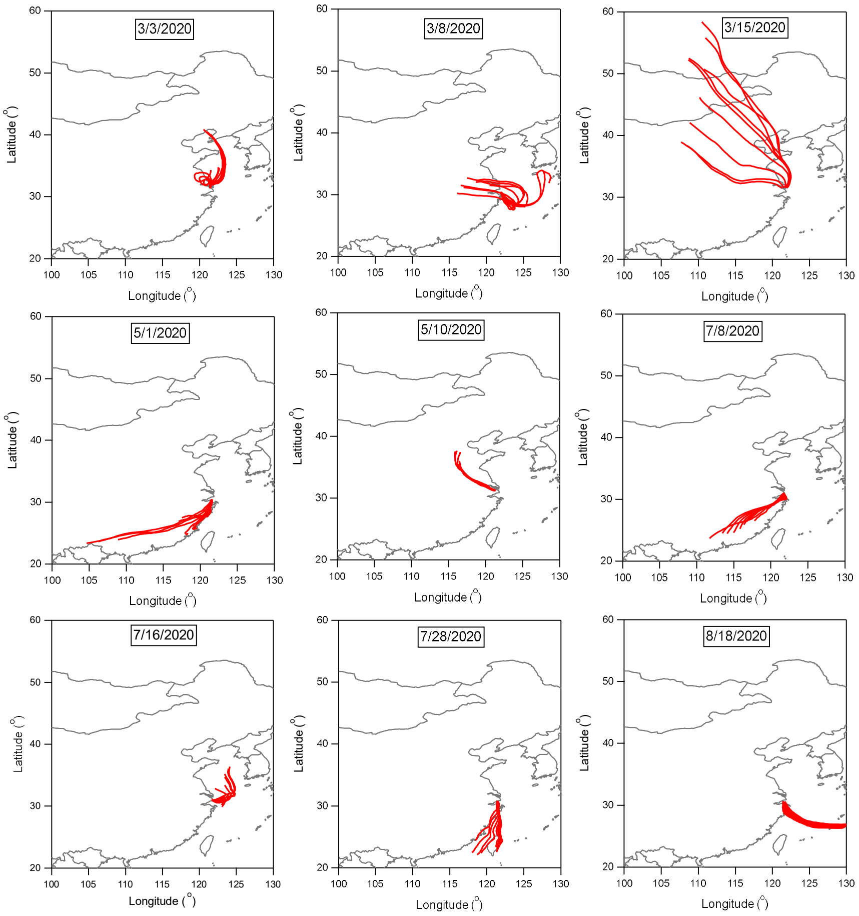

Figure S14. The 72-h backward trajectories arriving at the sampling site at an elevation of 500 m for the 9 days under Case#2 with A_0=0 for BaP decay.

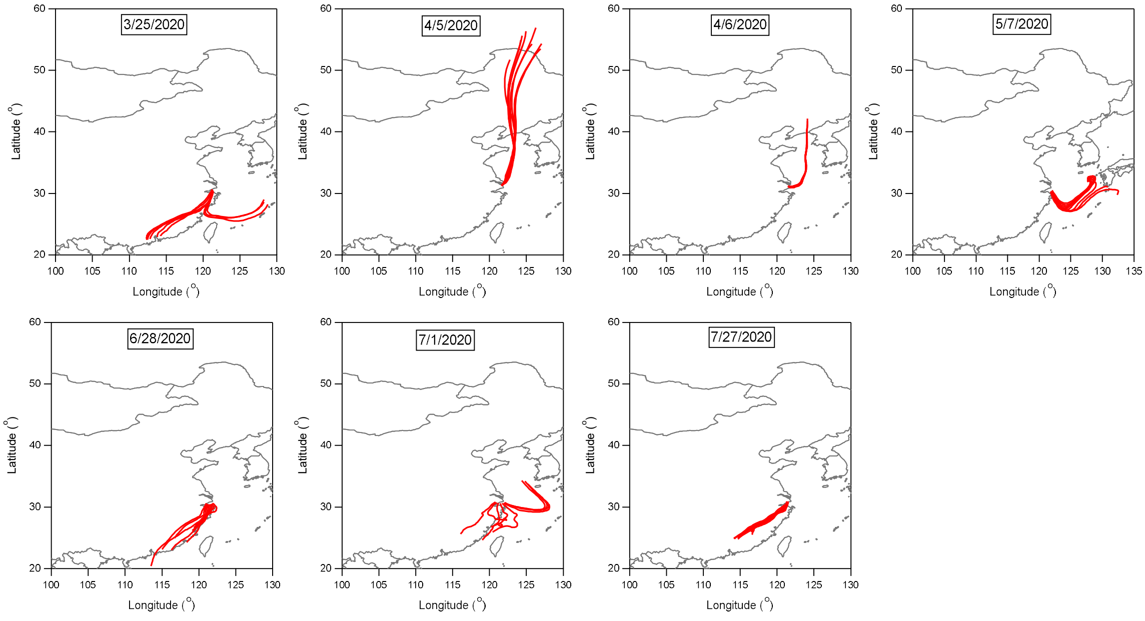

Figure S15. The 72-h backward trajectories arriving at the sampling site at an elevation of 500 m for the 7 days under Case#2 with A_0=0 for BaF decay.

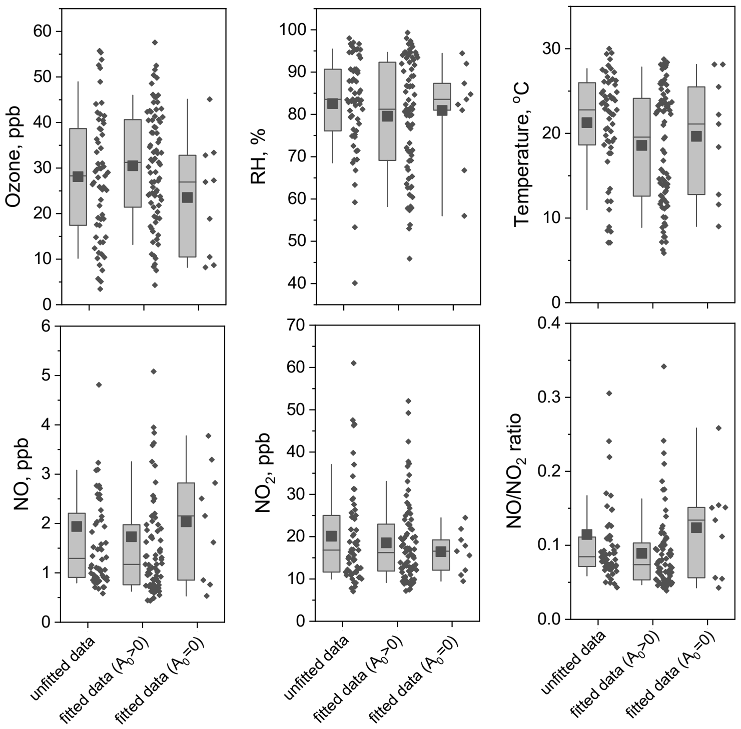

Figure S16. Box plot of the environmental parameters (O_3, NO, NO_2, NO/NO_2, RH, and T) for samples successfully fitted with the decay function and those that were not.

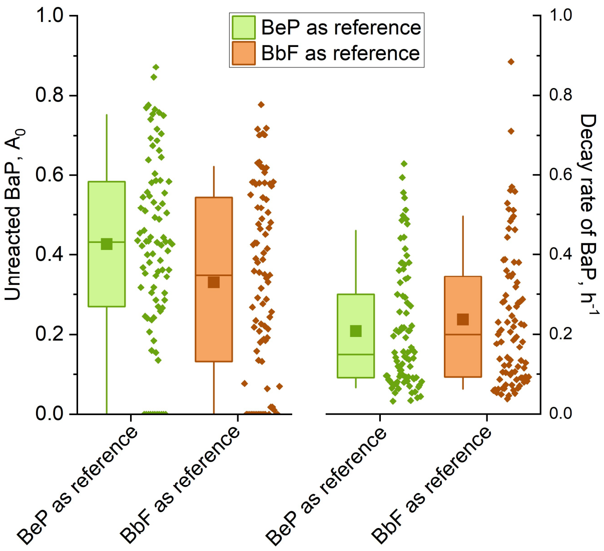

Figure S17. Comparisons of the BaP decay rates and unreacted fraction (A_0) derived using BeP as reference versus those using BbF as reference.

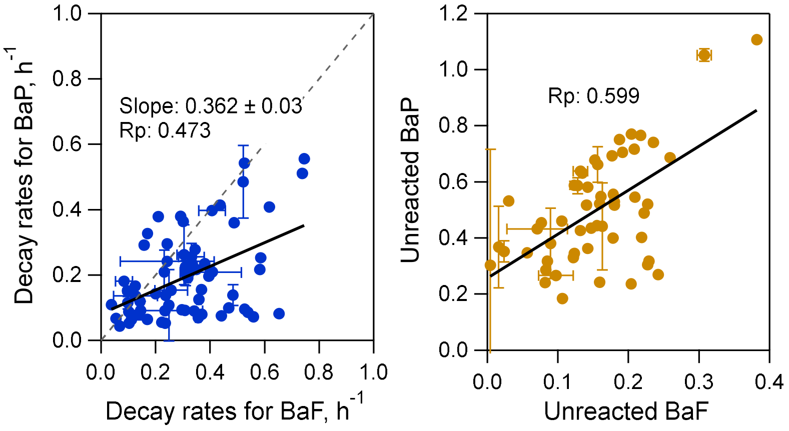

Figure S18. Comparisons of the BaP decay rates and unreacted fraction (A_0) versus those for BaF.

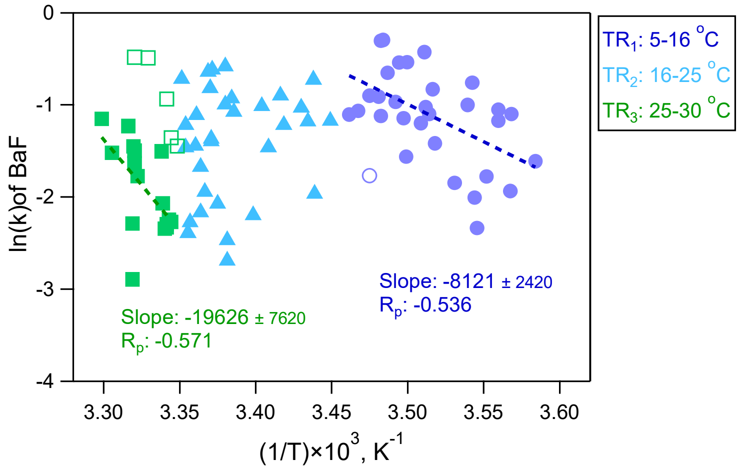

Figure S19. Arrhenius plot of the decay rates (k) of BaF with ambient temperature. Data are shown in different colors indicating different temperature ranges. Solid markers represent the data used for the linear fitting of ln(k) vs 1/T for each temperature ranges. The calculated slope is positively associated with the effective activation energy for each scenario.

Table S1. Statistical summary of measured PAH species in this study.

PAHs	Abbreviation	Formula	Molecular weight	Average (ng m^{-3})	Stdev (ng m^{-3})	Range (ng m^{-3})
Anthracene	ANT	$C_{14}H_{10}$	178	0.54	0.47	ND*-4.70
Phenanthrene	PHE	$C_{14}H_{10}$	178	0.43	0.24	ND-2.10
Fluoranthene	FLU	$C_{16}H_{10}$	202	0.26	0.17	ND-2.53
Pyrene	PYR	$C_{16}H_{10}$	202	0.46	0.25	ND-2.41
Cyclopenta[cd]pyrene	CPP	$C_{18}H_{10}$	226	0.11	0.12	ND-1.12
Benzo[c]phenanthrene	BcP	$C_{18}H_{12}$	228	0.13	0.11	ND-0.95
Benz[a]anthrancene	BaA	$C_{18}H_{12}$	228	0.10	0.10	ND-1.35
Benzo[b]fluoranthene	BbF	$C_{20}H_{12}$	252	0.29	0.23	ND-2.03
Benzo[k]fluoranthene	BkF	$C_{20}H_{12}$	252	0.18	0.14	ND-1.28
Benzo[a]fluoranthene	BaF	$C_{20}H_{12}$	252	0.05	0.04	ND-0.40
Benzo[e]pyrene	BeP	$C_{20}H_{12}$	252	0.27	0.21	ND-2.15
Benzo[a]pyrene	BaP	$C_{20}H_{12}$	252	0.20	0.17	ND-2.01
Perylene	PER	$C_{20}H_{12}$	252	0.28	0.24	ND-1.92
Benzo[ghi]perylene	BghiP	$C_{22}H_{12}$	276	0.34	0.29	ND-2.05
Indeno[123-cd-]pyrene	IcdP	$C_{22}H_{12}$	276	0.28	0.24	ND-1.95
Benzo[b]chrysene	BbC	$C_{22}H_{14}$	278	0.04	0.05	ND-0.40
Dibena[a,c]anthracene	DacA	$C_{22}H_{14}$	278	0.03	0.03	ND-0.27
Dibenzo[a,h]anthracene	DahA	$C_{22}H_{14}$	278	0.02	0.03	ND-0.25
Picene		$C_{22}H_{14}$	278	0.03	0.03	ND-0.19
Coronene	COR	$C_{24}H_{12}$	300	0.02	0.03	ND-0.22
**Total PAHs**	/	/	/	**4.02**	**2.38**	**0.21-20.17**

* not detected.

Table S2. Summary of decay kinetics of BaP heterogenous reaction with ozone from previous laboratory studies.

Reference	Substrate	O_3 (ppm)	T (K)	RH	K_{O3} (10^{-13} cm^3)[a]	K_{max} (s^{-1})[a]	Dependence on ozone
Kwamena et al.[10]	Azelaic acid aerosols (dry), solid organic aerosols	0-45	298	<1%	0.012 ± 0.0004	0.048 ± 0.008	Langmuir-Hinshelwood behavior
	Azelaic acid aerosols (wet), solid organic aerosols	0-36	298	~72%	0.028 ± 0.014	0.060 ± 0.018	Langmuir-Hinshelwood behavior
	Dry NaCl aerosols, salt aerosols	0-31	298	<1%	no reaction was observed		
Poschl et al.[11]	spark discharge Soot aerosol particles	0-1	298	<1%, 25%	2.8 ± 0.2	0.015 ± 0.001	Langmuir-Hinshelwood behavior
Zhou et al.[12]	ammonium sulfate (AS) particles	3.6-32	296	<5%	0.14 ± 0.04	0.034 ± 0.002	Langmuir-Hinshelwood behavior
	AS coated with BES (bis(2-ethylhexyl) sebacate, liquid)				0.041 ± 0.001	0.051 ± 0.001	Langmuir-Hinshelwood behavior
	AS coated with PSO (phenyl-siloxane oil, liquid)				0.13 ± 0.04	0.047 ± 0.004	Langmuir-Hinshelwood behavior
Zhou et al.[13]	AS-thin SOA dry	1.5-28	296	<5%	0.051 ± 0.009	0.042 ± 0.004	Langmuir-Hinshelwood behavior
	AS-thick SOA dry		296	<5%	0.023 ± 0.004	0.022 ± 0.003	
	AS-SOA 50% RH		296	50%	0.14 ± 0.044	0.023 ± 0.004	
	AS-SOA 70% RH		296	70%	0.062 ± 0.011	0.039 ± 0.004	
**Reference**	**Substrate**	**O_3 (ppm)**	**T (K)**	**RH**	**A (10^{-3} s^{-1})[b]**	**B (10^{15} cm^{-3})[b]**	**Dependence on ozone**
Kahan et al.[14]	Octanol film	14-935	298	<1%	5.5 ± 0.2	2.8 ± 0.4	Langmuir-Hinshelwood behavior
**Reference**	**Substrate**	**O_3 (ppm)**	**T (K)**	**RH**	**k_2 (ppm^{-1} min^{-1})[c]**		**Dependence on ozone**
Wu et al.[15]	fused silica plates	0-1.5	298	<1%	1.3 ± 0.2		Linear dependence
Alebic-Juretic et al.[16]	nonactivated silica gel, lower than monolayer coverage	0.05-0.4	298	<1%	0.449 ± 0.041		Linear dependence
	nonactivated silica gel, higher than monolayer coverage				0.190 ± 0.002		
Perraudin et al.[17]	graphite (carbonaceous particles)	1.6-13	295	<1%	0.078 ± 0.022		Linear dependence
	silica (mineral)	1.6-13	295	<1%	0.207 ± 0.044		

[a] the decay rate k can be obtained by this equation $k = \frac{k_{max} \cdot K_{O_3} \cdot [O_3(g)]}{1 + K_{O_3} \cdot [O_3(g)]}$; [b] the decay rate k can be obtained by this equation $k = \frac{A \cdot [O_3(g)]}{B + [O_3(g)]}$. [c] the decay rate k can be obtained by this equation $k = k_2 \cdot [O_3(g)]$

References

(1) Wang, Q.; Yu, J. Z. Ambient Measurements of Heterogeneous Ozone Oxidation Rates of Oleic, Elaidic, and Linoleic Acid Using a Relative Rate Constant Approach in an Urban Environment. *Geophys. Res. Lett.* **2021**, *48* (19), e2021GL095130. https://doi.org/10.1029/2021GL095130.

(2) Wang, Q.; Wang, S.; Chen, H.; Zhang, Z.; Yu, H.; Chan, M. N.; Yu, J. Z. Ambient Measurements of Daytime Decay Rates of Levoglucosan, Mannosan, and Galactosan. *J. Geophys. Res. Atmos.* **2025**, *130* (8), e2024JD042423. https://doi.org/10.1029/2024JD042423.

(3) Huff Hartz, K. E.; Weitkamp, E. A.; Sage, A. M.; Donahue, N. M.; Robinson, A. L. Laboratory Measurements of the Oxidation Kinetics of Organic Aerosol Mixtures Using a Relative Rate Constants Approach. *J. Geophys. Res. Atmos.* **2007**, *112* (4), 1–13. https://doi.org/10.1029/2006JD007526.

(4) Nielsen, T. Reactivity of Polycyclic Aromatic Hydrocarbons toward Nitrating Species. *Environ. Sci. Technol.* **1984**, *18* (3), 157–163. https://doi.org/10.1021/es00121a005.

(5) Zimmermann, K.; Jariyasopit, N.; Massey Simonich, S. L.; Tao, S.; Atkinson, R.; Arey, J. Formation of Nitro-PAHs from the Heterogeneous Reaction of Ambient Particle-Bound PAHs with N2O5/NO3/NO 2. *Environ. Sci. Technol.* **2013**, *47* (15), 8434–8442. https://doi.org/10.1021/es401789x.

(6) Paatero, P.; Tapper, U. Positive Matrix Factorization: A Non-negative Factor Model with Optimal Utilization of Error Estimates of Data Values. *Environmetrics* **1994**, *5* (2), 111–126. https://doi.org/10.1002/env.3170050203.

(7) Reff, A.; Eberly, S. I.; Bhave, P. V. Receptor Modeling of Ambient Particulate Matter Data Using Positive Matrix Factorization: Review of Existing Methods. *J. Air Waste Manag. Assoc.* **2007**, *57* (2), 146–154. https://doi.org/10.1080/10473289.2007.10465319.

(8) Wang, Q.; Qiao, L.; Zhou, M.; Zhu, S.; Griffith, S.; Li, L.; Yu, J. Z. Source Apportionment of PM2.5 Using Hourly Measurements of Elemental Tracers and Major Constituents in an Urban Environment: Investigation of Time-Resolution Influence. *J. Geophys. Res. Atmos.* **2018**, *123* (10), 5284–5300. https://doi.org/10.1029/2017JD027877.

(9) Zhu, S.; Wang, Q.; Qiao, L.; Zhou, M.; Wang, S.; Lou, S.; Huang, D.; Wang, Q.; Jing, S.; Wang, H.; Chen, C.; Huang, C.; Yu, J. Z. Tracer-Based Characterization of Source Variations of PM2.5and Organic Carbon in Shanghai Influenced by the COVID-19 Lockdown. *Faraday Discuss.* **2021**, *226*, 112–137. https://doi.org/10.1039/d0fd00091d.

(10) Kwamena, N. O. A.; Thornton, J. A.; Abbatt, J. P. D. Kinetics of Surface-Bound Benzo[a]Pyrene and Ozone on Solid Organic and Salt Aerosols. *J. Phys. Chem. A* **2004**, *108* (52), 11626–11634. https://doi.org/10.1021/jp046161x.

(11) Poschl, U.; Letzel, T.; Schauer, C.; Niessner, R. Interaction of Ozone and Water Vapor with Spark Discharge Soot Aerosol Particles Coated with Benzo[a]Pyrene Degradation, and Atmospheric Implications. *J. Phys. Chem. A* **2001**, *105*, 4029–4041.

(12) Zhou, S.; Lee, A. K. Y.; McWhinney, R. D.; Abbatt, J. P. D. Burial Effects of Organic Coatings on the Heterogeneous Reactivity of Particle-Borne Benzo[a]Pyrene (BaP) toward Ozone. *J. Phys. Chem. A* **2012**,

116 (26), 7050–7056. https://doi.org/10.1021/jp3030705.

(13) Zhou, S.; Shiraiwa, M.; McWhinney, R. D.; Pöschl, U.; Abbatt, J. P. D. Kinetic Limitations in Gas-Particle Reactions Arising from Slow Diffusion in Secondary Organic Aerosol. *Faraday Discuss.* **2013**, *165*, 391–406. https://doi.org/10.1039/c3fd00030c.

(14) Kahan, T. F.; Kwamena, N. O. A.; Donaldson, D. J. Heterogeneous Ozonation Kinetics of Polycyclic Aromatic Hydrocarbons on Organic Films. *Atmos. Environ.* **2006**, *40* (19), 3448–3459. https://doi.org/10.1016/j.atmosenv.2006.02.004.

(15) Wu, C. H.; Salmeen, I.; Niki, H. Fluorescence Spectroscopic Study of Reactions between Gaseous Ozone and Surface-Adsorbed Polycyclic Aromatic Hydrocarbons. *Environ. Sci. Technol.* **1984**, *18* (8), 603–607. https://doi.org/10.1021/es00126a007.

(16) Alebic-Juretic, A.; Cvitas, T.; Klasinc, L. Heterogeneous Polycyclic Aromatic Hydrocarbon Degradation with Ozone on Silica Gel Carrier. *Environ. Sci. Technol.* **1990**, *24* (1), 62–66. https://doi.org/10.1021/es00071a005.

(17) Perraudin, E.; Budzinski, H.; Villenave, E. Kinetic Study of the Reactions of Ozone with Polycyclic Aromatic Hydrocarbons Adsorbed on Atmospheric Model Particles. *J. Atmos. Chem.* **2007**, *56* (1), 57–82. https://doi.org/10.1007/s10874-006-9042-x.
